# The 12 o’clock assay: an optimized dodecaplex droplet digital PCR assay for robust DNA methylation quantification and epigenetic clock-based age-predictions

**DOI:** 10.1186/s13148-026-02105-0

**Published:** 2026-03-24

**Authors:** Ilef Hchaichi, Imène Garali, Alina-Madalina Popa, Antoine Daunay, Nicolas P. Tessier, Mourad Sahbatou, Lise M. Hardy, Aurore Rampanou, Hélène Blanché, Jean-François Zagury, Mathilde Touvier, Hélène Le Buanec, Shufang Renault, Nicolas Girerd, Jean-François Deleuze, Alexandre How-Kit

**Affiliations:** 1 Laboratory for Genomics, Foundation Jean Dausset – CEPH, 75010 Paris, France; 2 PSE-SANTE/SERAMED/LRAcc, Autorité de Sûreté Nucléaire et de Radioprotection (ASNR), 92260 Fontenay-Aux-Roses, France; 3 Laboratory for Bioinformatics, Foundation Jean Dausset – CEPH, Paris, France; 4 Circulating Tumor Biomarkers Laboratory, Inserm CIC-BT 1428, Department of Translational Research, Institut Curie, Paris, France; 5 CEPH-Biobank, Foundation Jean Dausset - CEPH, Paris, France; 6 Équipe Génomique, Bioinformatique et Chimie Moléculaire (EA 7528), Conservatoire National des Arts et Métiers, Paris, France; 7 INSERM, INRAE, CNAM, Centre for Research in Epidemiology and StatisticS (CRESS), Nutritional Epidemiology Research Team (EREN), Université Sorbonne Paris Nord and Université Paris Cité, 93017 Bobigny, France; 8 INSERM U976 - HIPI Unit, Saint-Louis Research Institute, University of Paris, Paris, France; 9 Centre d’Investigation Clinique Plurithématique 1433 and Inserm U1116, CHRU Nancy, F-CRIN INI-CRCT (Cardiovascular and Renal Clinical Trialists), Université de Lorraine, Nancy, France; 10 Centre National de Recherche en Génomique Humaine, CEA, Institut François Jacob, Evry, France

**Keywords:** DNA methylation, ddPCR, Age-prediction model, Epigenetic clock, Machine-learning, Biological age

## Abstract

DNA methylation quantification in age-prediction models relies on only a few techniques with inherent limitations and biases, reducing the accuracy and reproducibility. We developed a new droplet digital PCR assay format for accurate DNA methylation quantification, based on three LNA-containing hydrolysis probes to detect methylated and unmethylated alleles. Using a 12-plex assay we simultaneously quantified methylation at six CpGs (*ASPA*, *C1orf132*, *CCDC102B*, *EDARADD*, *ELOVL2*, *FHL2*) in 351 blood samples (age range: 0–95 years), showing strong correlations with age (|*r|*> 0.83). Several age-prediction models were developed, achieving high performance on testing sets (*R*^2^: 0.933–0.973, MAE: 3.18–5.18, RMSE: 4.71–7.27).

**Supplementary Information:**

The online version contains supplementary material available at 10.1186/s13148-026-02105-0.

## Introduction

The epigenetic clock describes DNA methylation changes that consistently vary with age across individuals [1]. It enabled the identification of DNA methylation-based biomarkers that are strongly correlated with chronological age, leading to the development of age-prediction models applicable to multiple tissues, most notably blood. These models are also known as epigenetic clocks (hereafter referred to as e-clocks). To date, numerous e-clocks have been developed classified according to their generation [2]. First- and second-generation e-clocks are primarily designed to predict the chronological age and biological age, respectively [1]. Third-generation e-clocks have been proposed to measure the rate of aging [3]. They have numerous applications, including the accurate estimation of a sample’s chronological age [4], and the assessment of epigenetic aging and/or biological age in relation to various phenotypes [1].

While several DNA methylation-based biomarkers (i.e. CpGs) and mathematical methods have been implemented in e-clocks [1, 5], only few technologies are currently used to quantify DNA methylation. Epigenotyping microarrays (Illumina BeadChips) are widely used for DNA methylation quantification in e-clocks covering from a few dozens to over 1,000 CpGs [1, 2]. However, they do not allow for highly accurate quantification of DNA methylation, presenting an analytical resolution around 10–15% (Table S1). On the other hand, e-clocks based on a small number of CpGs (from two to over a dozen) rely on quantitative DNA methylation technologies, offering improved accuracy and resolution compared to microarrays (Table S1). Pyrosequencing is one the most widely used approach, but we can also cite amplicon sequencing, SNaPshot™ and EpiTYPER© [5–7]. However, they also present several limitations (Table S1), including difficulties for multiplexing. This complicate the implementation, validation and application of published e-clocks to independent cohorts, due to technical issues and inter-laboratory variability [8–10].

Since 2014, droplet digital PCR (ddPCR) emerged as a new technology for DNA methylation quantification [11]. Application of Poisson statistics to ddPCR allows sensitive, accurate, and absolute quantification of DNA molecules, decreasing the biases encountered with other methods [12]. Here we developed an optimized 12-plex ddPCR assay detecting both methylated and unmethylated alleles per locus within a single fluorescence channel, using six colors in total. The assay enables robust DNA methylation quantification at six age-related CpGs simultaneously and was named the 12 o’clock assay, as it was designed for epigenetic clock-based age prediction. It allows the development of several optimized e-clocks whose performances were compared.

## Methods

Blood and DNA samples: a total of 351 blood/buffy-coat-derived DNA samples from unrelated French individuals across four different cohorts were used (see Supplementary Materials, Table S2, and Figure S1).

Primer and probe design (Tables S3 and S4), bisulfite conversion, PCR, and pyrosequencing procedures are provided in the Supplementary Methods.

Multiplex ddPCR: from 0.625 ng to 40 ng of converted DNA was used as template in multiplex ddPCR experiments performed on a QX600™ ddPCR™ System (Bio-Rad). The reaction mix was prepared in 96-well PCR plates and included 300–900 nM of forward and reverse primers and 85–300 nM of probes depending on the assay (Table S3), 5.5 µl of DNA and 1 X of ddPCR Multiplex Supermix (Bio-Rad) in a total volume of 22 µl, then partitioned into approximately 20,000 nanoliter-sized droplets per well using the AutoDG (Bio-Rad). PCR amplification was performed using a MasterCycler ProS (Eppendorf). The lid was set to 105 °C and the sample volume to 40 µl. PCR cycling included an initial denaturation step at 95 °C for 10 min, followed by 50 2-step cycles including 45 s denaturation at 94 °C and 1 min primer annealing and elongation at 58 °C (2 °C per second ramp rate). The final step included 10 min at 98 °C, followed by cooling to 4 °C indefinitely. The droplets were read by the QX600 Droplet Reader (Bio-Rad). Data were analyzed with QX Manager™ software 2.1.0 using 1D and 2D amplitude plots, and two thresholds: one to quantify methylated (*CCDC102B* and *ELOVL2*) or unmethylated (*ASPA*, *C1orf132*, *EDARADD* and *FHL2*) droplets, and another for the total positive droplets (Figure S2). CpG methylation was calculated as follow: $\frac{\text{Methylated\, droplets}}{\text{Methylated }+\text{ Unmethylated \,droplets}}$ for *CCDC102B* and *ELOVL2* or $1-\frac{\text{Unmethylated \,droplets}}{\text{Methylated }+\text{ Unmethylated \,droplets}}$ for *ASPA*, *C1orf132*, *EDARADD* and *FHL2*.

Predictive model development: DNA methylation-based age prediction models were developed using elastic net regression (ENR), gradient boosting regressor (GBR), support vector machines, and Klemera-Doubal regression (KDR), as detailed in Supplementary Methods. Model performance was assessed using Pearson’s *r*, *R*^2^, mean absolute error (MAE), and root mean square error (RMSE) between predicted and chronological ages. All the data and source code are available in the Supplementary Materials.

## Results

To develop an amplitude-based multiplex ddPCR assay capable of quantifying DNA methylation at six age-associated CpG sites simultaneously, we selected six CpG sites within the *ASPA*, *C1orf132*, *CCDC102B*, *EDARADD*, *ELOVL2*, and *FHL2* loci (Figure S3A), previously used in several age-prediction models [8]. The detection strategy relied on three probes per locus (Figure S3B): two fluorescent-labeled probes targeting a locus-specific, methylation-independent region and either the methylated or unmethylated CpG, and a third unlabeled (invisible) probe targeting the complementary methylation state. Using the QX600 ddPCR system, we were able to detect both methylated and unmethylated alleles at six CpG sites.

**Fig. 1:**
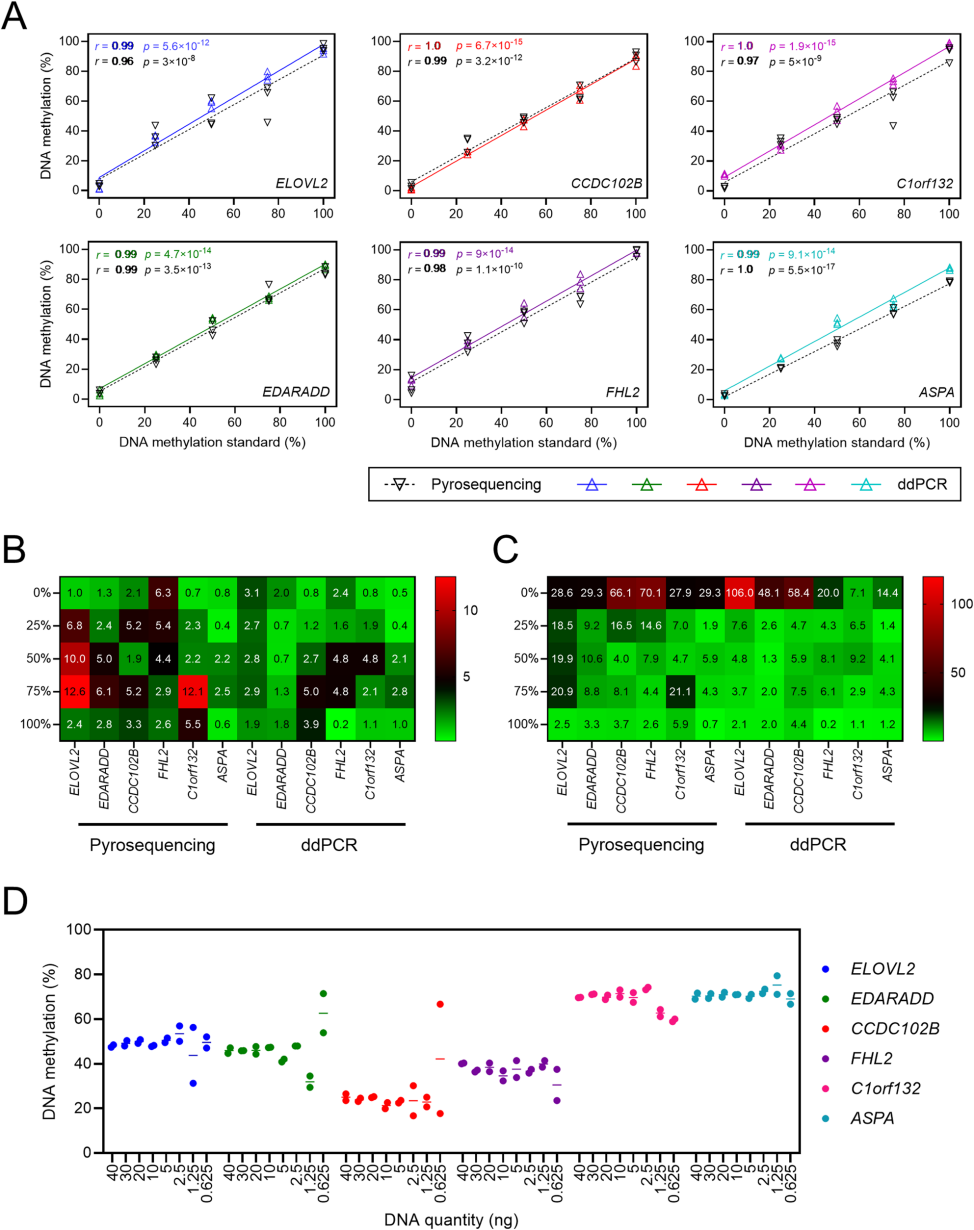
Assessment of the reproducibility and robustness of DNA methylation measurements using the 12 o’clock assay. **A** Correlation analysis of DNA methylation values at the six CpG sites measured by ddPCR and pyrosequencing using DNA methylation standards. Pearson’s *r* coefficients and *p*-values are shown. **B, C** Heatmap representation of the standard deviation (SD) (**B**) and coefficient of variation (CV, %) (**C**) obtained from replicate measurements of DNA methylation of the standards using ddPCR and pyrosequencing. **A–C** Triplicate experiments were performed using 10 ng of DNA methylation standards per ddPCR and PCR reaction. **D** Evaluation of DNA methylation quantification at the six CpG sites using the 12 o’clock assay with decreasing amounts of bisulfite-converted whole blood DNA (from 40 to 0.625 ng, Promega). Duplicate experiments were performed for each DNA input. Individual values are shown as dots, and the mean of duplicate measurements is represented as a bar

The assay was evaluated on 0%, 25%, 50%, 75%, and 100% DNA methylation standards, yielding two positive droplet clusters corresponding to methylated and unmethylated alleles at each locus, as expected (Figure S4A and S4B). DNA methylation values were close to expected values, with reduced variation across replicates and slightly better correlations compared to pyrosequencing (Fig. 1A–C), highlighting the reproducibility of the ddPCR assay. Since DNA methylation is calculated directly from counts of positive and high-positive droplets, the number of positive droplets per CpG should remain within the limiting dilution range. We further tested assay sensitivity using decreasing amounts of bisulfite-treated commercial whole-blood DNA (40–0.625 ng; Figs. 1D and S5). For each CpG, the number of positive droplets decreased from 2,482 to 13, corresponding to 13.17% to 0.08% of positive droplets, with 93.11% to 99.96% of them originating from a single DNA molecule per target (Figure S5). Methylation levels remained stable across duplicates and down to ~ 5 ng input, but became more variable at lower concentrations (Fig. 1D). For optimal, reproducible, and robust assay performance, we recommend maintaining ≤ 10% positive droplets per target CpG (5–30 ng of converted DNA as template), ensuring ≥ 95% single-molecule occupancy after partitioning.

The assay was further applied to blood-derived DNA from 351 individuals of the French general population aged 0–95 years at collection (Table S2). Across all samples, DNA methylation levels were strongly and significantly correlated with age at each CpG site (*p* < 9.7 × 10⁻⁸⁹): positively for *ELOVL2* (*r* = 0.85) and *FHL2* (*r* = 0.93), and negatively for *ASPA* (*r* =  − 0.83), *C1orf132* (*r* =  − 0.93), *CCDC102B* (*r* =  − 0.83), and *EDARADD* (*r* =  − 0.88) (Figure S6A). Correlations were highly similar between male and female samples, indicating no sex bias (Figure S7). For three CpGs (*ELOVL2*, *EDARADD*, and *CCDC102B*), methylation did not follow a strictly linear relationship with age, so we transformed them to improve the linear correlation from 0.85, − 0.88, and − 0.83 to 0.87, − 0.89, and − 0.91, respectively (Figure S6B).

Age-prediction models were developed based on ENR, GBR, SVM_l_, SVM_r_, and KDR, using either raw or transformed DNA methylation data from the six CpGs. Using raw DNA methylation data, the model with the best prediction performance on the training set was GBR (*r* = 0.9996, *R*^2^ = 0.999, MAE = 0.54, RMSE = 0.72), followed by KDR, SVM_r_, SVM_l_, and ENR. On the testing set, KDR performed best (*r* = 0.9863, *R*^2^ = 0.973, MAE = 3.51, RMSE = 4.72), followed by GBR, SVM_r_, ENR, and SVM_l_ (Figure S8A, Table 1). With transformed DNA methylation data, GBR remained the top-performing model on the training set (*r* = 0.9996, *R*^2^ = 0.999, MAE = 0.53, RMSE = 0.73), followed by KDR, SVM_r_, SVM_l_, and ENR, while KDR again achieved the best performance on the testing set (*r* = 0.9866, *R*^2^ = 0.973, MAE = 3.18, RMSE = 4.71), followed by GBR, SVM_r_, SVM_l_, and ENR (Figure S8A, Table 1). These performances were confirmed on an independent cohort (n = 116, ages 38–61 years, Figure S9 and Table 1), showing similar MAE and RMSE despite lower *r* and *R*^2^ due to the narrower age range [13], outperforming previous pyrosequencing-based age predictions (Table S5) [14].

**Table 1:** Age prediction performances of the different epigenetic clocks based on the six CpG sites

Statistical models			Estimators			
Data type^a^	Name	Dataset^b^	*r*	*R*^2^	MAE	RMSE
Raw	ENR	Training	0.9659	0.933	5.30	6.96
		Testing	0.9645	0.930	5.33	7.43
		Training (38–61 years)	0.6377	0.407	5.74	7.06
		Independent Testing	0.5800	0.336	4.59	5.9
	GBR	Training	0.9996	0.999	0.54	0.72
		Testing	0.9699	0.941	4.47	6.85
		Training (38–61 years)	0.9947	0.989	0.53	0.70
		Independent Testing	0.5293	0.280	5.25	6.29
	SVM_l_	Training	0.9654	0.932	5.23	7.04
		Testing	0.9645	0.930	5.21	7.46
		Training (38–61 years)	0.6459	0.417	5.58	6.97
		Independent Testing	0.5797	0.336	4.63	5.92
	SVM_r_	Training	0.9774	0.955	4.02	5.70
		Testing	0.9703	0.942	4.54	6.86
		Training (38–61 years)	0.6672	0.445	4.44	5.84
		Independent Testing	0.5735	0.329	5.47	6.63
	KDR	Training	0.9867	0.974	3.32	4.43
		Testing	0.9863	0.973	3.51	4.72
		Training (38–61 years)	0.8195	0.672	3.59	4.45
		Independent Testing	0.7874	0.620	2.91	3.67
Transformed	ENR	Training	0.9695	0.940	4.93	6.59
		Testing	0.9661	0.933	5.18	7.27
		Training (38–61 years)	0.6374	0.406	5.25	6.50
		Independent Testing	0.5885	0.346	4.81	5.96
	GBR	Training	0.9996	0.999	0.53	0.73
		Testing	0.9696	0.940	4.46	6.87
		Training (38–61 years)	0.9946	0.989	0.53	0.71
		Independent Testing	0.5461	0.298	5.36	6.39
	SVM_l_	Training	0.9690	0.939	4.88	6.65
		Testing	0.9667	0.935	4.90	7.22
		Training (38–61 years)	0.6521	0.425	5.02	6.28
		Independent Testing	0.5957	0.355	4.91	6.03
	SVM_r_	Training	0.9779	0.956	3.95	5.64
		Testing	0.9694	0.940	4.73	7.01
		Training (38–61 years)	0.6674	0.445	4.40	5.83
		Independent Testing	0.5810	0.338	4.94	6.11
	KDR	Training	0.9877	0.976	3.08	4.25
		Testing	0.9866	0.973	3.18	4.71
		Training (38–61 years)	0.8244	0.680	3.31	4.24
		Independent Testing	0.7864	0.618	2.87	3.68

^a^Data type indicates the type of DNA methylation data used for each model. For the "transformed" data, only DNA methylation values of *ELOVL2*, *EDARADD*, and *CCDC102B* have been transformed, while all six CpG methylation levels have been standardized prior to model construction (see Methods)

^b^The Training (38–61 years) dataset corresponds to prediction performance restricted to the same age range as the independent testing set (SU.VI.MAX) to enable direct comparison

Using transformed data improved the accuracy of models assuming a linear relationship between predictors and age (ENR, SVM_l_, and KDR), while having little effect on models less sensitive to linearity (GBR and SVM_r_). Differences between predicted and chronological ages using raw data revealed underestimations in newborns (ENR, SVM_l_, KDR) and nonagenarians (ENR, GBR, SVM_l_, SVM_r_), as well as slight overestimations in newborns for GBR and SVM_r_ (Figure S8B). These biases were largely corrected using transformed data in ENR-, SVM_l_-, KDR-based models, but persisted in GBR- and SVM_r_-based models.

Finally, we evaluated whether different combinations of the six CpGs (from two to five) could improve age prediction performance. Age prediction generally improved with more CpGs, with six-CpG e-clocks performing best, although a few reduced models performed similarly or slightly better, especially in the testing set or with the KDR model (Figure S10 and Tables S6 and S7).

## Discussion

Although numerous e-clocks have been published, most improvements concern either their dedicated applications [1] or, occasionally, the underlying mathematical approach used for age prediction [15], while changes in the methodology for DNA methylation quantification remain rare. DNA methylation measurement technologies remain largely restricted to a few standard methods, which all present limitations in terms of resolution and accuracy (Table S1).

Therefore, the 12 o’clock assay was designed to overcome these technical issues by leveraging ddPCR for absolute and accurate DNA quantification at six loci in a single reaction, an achievement not previously reported. Although a few studies have applied ddPCR to e-clocks, reporting increased accuracy, they were limited in the number of loci due to lower multiplexing capability [6, 16]. Standard ddPCR methylation detection uses either different fluorescent probes for alleles [6, 16] or two PCR assays (one for the methylated allele, one for the reference gene) [17]. While the first halves the number of multiplexable targets [6, 16], the second needs internal controls that may be sensitive to DNA degradation or copy number variations, potentially affecting results [17, 18]. In a recently published multiplex assay based on the first approach targeting four genes, a single fluorescence channel was used for one allele from each of two different genes leading to complex data analysis due to additional clusters under certain conditions [19]. This is avoided in our assay, as only one color is used per locus.

Limitations of our assay includes (i) the need for DNA at limiting dilution, and (ii) occasional ‘rain’ artifacts, where upper positive droplets fall into the lower cluster, affecting methylation accuracy. While the first is easy to manage by quantifying the converted DNA sample, the second was partially mitigated by labeling the probe targeting the rarest allele per locus in the population based on our previous pyrosequencing data [8]. However, high variability in methylation across ages makes this challenging. These artifacts remain a potential drawback, especially with degraded or low-quality DNA.

Regarding the developed e-clocks, which performed well, slight biases in age predictions were observed for the youngest and/or oldest individuals in most models. They could be attributed to (i) regression toward the mean (all models except KDR) [20] and/or (ii) nonlinear CpG methylation-age relationship in models assuming linearity (ELN, SVM_l_, and KDR). While transforming DNA methylation data reduced nonlinear biases in ELR-, SVM_l_-, and KDR-based models, the regression-to-the-mean effect could not be directly eliminated. Therefore, relative age acceleration is often preferred over absolute age acceleration for group comparisons to account for this effect [15], except with KDR-based models or age-matched groups.

In conclusion, our optimized ddPCR assay is robust, cost-effective (~ $15 per sample), easy-to-handle, with a time-to-result of ~ 4 and ~ 6 h for 8 and 96 samples, respectively. The models developed enable different applications and should be selected depending on the user’s objective. Forensic applications should focus on models with the highest prediction accuracy regardless of the number of CpGs or the methods used (noting that KDM models require chronological age as input). As they may capture a broader range of epigenetic variation, models including all six CpGs may be preferred for studies on biological age or epigenetic aging, using performance within the age range of interest as a key selection criterion.

## Supplementary Information

Below is the link to the electronic supplementary material.

Supplementary Material 1.

## Data Availability

All data generated or analyzed during this study are included in this published article and its supplementary information files.
