# Supplementary material for: The 12 o’clock assay: an optimized dodecaplex droplet digital PCR assay for robust DNA methylation quantification and epigenetic clock-based age-predictions
Source: Clin Epigenetics. 2026 Mar 24;18:78. doi: 10.1186/s13148-026-02105-0 (PMC13134083; doi:10.1186/s13148-026-02105-0)
Supplement: Supplementary file 1 — Supplementary Material 1. [file 13148_2026_2105_MOESM1_ESM.pdf]

Supplementary data for: The 12 o'clock assay: an optimized dodecaplex droplet digital PCR assay for robust DNA methylation quantification and epigenetic clock-based age-predictions

Ilef Hchaichi[1], Imène Garali[2], Alina-Madalina Popa[1], Antoine Daunay[1], Nicolas P. Tessier[1], Mourad Sahbatou[3], Lise M. Hardy[1], Aurore Rampanou[4], Hélène Blanché[5], Jean-François Zagury[6], Mathilde Touvier[7], Hélène Le Buanec[8], Shufang Renault[4], Nicolas Girerd[9], Jean-François Deleuze[1,3,5,10] & Alexandre How-Kit[1‡]

[1] Laboratory for Genomics, Foundation Jean Dausset – CEPH, Paris, France

[2] Autorité de sûreté nucléaire et de radioprotection (ASNR), PSE-SANTE/SERAMED/LRAcc, F-92260, Fontenay-aux-Roses, France

[3] Laboratory for Bioinformatics, Foundation Jean Dausset – CEPH, Paris, France

[4] Circulating Tumor Biomarkers Laboratory, Inserm CIC-BT 1428, Department of Translational Research, Institut Curie, Paris, France

[5] CEPH-Biobank, Foundation Jean Dausset - CEPH, Paris, France

[6] Équipe Génomique, Bioinformatique et Chimie Moléculaire (EA 7528), Conservatoire National des Arts et Métiers, Paris, France

[7] Université Sorbonne Paris Nord and Université Paris Cité, INSERM, INRAE, CNAM, Centre for Research in Epidemiology and StatisticS (CRESS), Nutritional Epidemiology Research Team (EREN), F-93017 Bobigny, France

[8] INSERM U976 - HIPI Unit, Saint-Louis Research Institute, University of Paris, Paris, France

[9] Université de Lorraine, Centre d'Investigation Clinique Plurithématique 1433 and Inserm U1116, CHRU Nancy, F-CRIN INI-CRCT (Cardiovascular and Renal Clinical Trialists), Nancy, France

[10] Centre National de Recherche en Génomique Humaine, CEA, Institut François Jacob, Evry, France

[‡] ***Correspondence to:***

Alexandre How-Kit, Ph.D., Laboratory for Genomics, Foundation Jean Dausset - CEPH, Paris, F-75010, France, Tel.: +33-(0)1- 53725146, email: ahowkit@cephb.fr

Supplementary Materials and methods

Human blood and DNA samples

The study was approved by three institutional review boards (CCPPRB Saint-Antoine, CCP Est III—Nancy, and Institutional Review Board No 00006477 of Paris North Hospitals) and conducted in accordance with current ethical and legal frameworks. All methods were performed following the recommendations of the French National Committee of Ethics. Blood and DNA samples from 351 unrelated French individuals from four different cohorts were used in this study (Table S2, Figure S1). 17 cord blood samples from newborns were provided by the Institut Saint-Louis, 40 blood-extracted DNA samples of children and teenagers aged from 5 to 17 years from the STANISLAS cohort (1) were provided by the CRB LORRAIN and CIC-P de Nancy, 196 blood samples from healthy donors aged from 19 to 66 years were provided by the Etablissement Français du Sang (EFS, Research Agreement 15/EFS/012) and 98 blood-extracted DNA samples from the participants of the CEPH Aging cohort (2) aged from 55 to 95 were provided by the CEPH Biobank. For blood samples, DNA extraction was performed on buffy coats using the QIAmp DNA blood mini Kit (Qiagen) according to the manufacturer's instructions. DNA was quantified using the Qubit™ dsDNA HS assay Kit on a Qubit 3 Fluorometer (Thermo Fischer Scientific) according to the manufacturer's instructions. An independent set of bisulfite-treated, buffy coat–derived DNA samples from 116 French individuals aged 38–61 years from the SU.VI.MAX cohort, previously analyzed by pyrosequencing (3), was provided for independent validation experiments.

Primers and probes design

All ddPCR/PCR primers and hydrolysis probes were first designed using Beacon Designer 8 (PREMIER Biosoft), and then manually optimized (Table S3). Pyrosequencing primers were either newly designed using the SNP Primer design software (Qiagen), or taken from previously published studies (Table S4). All primers and probes were ordered from Eurogentec. Modified and unmodified oligonucleotides were purified using (RP-)HPLC and RP-Gold, respectively.

Bisulfite conversion & quantification of bisulfite-treated DNA

Bisulfite conversion of DNA was performed on 800–1500 ng of genomic DNA, using the EpiTect Fast 96 DNA Bisulfite Kit (Qiagen) according to the manufacturer's instructions. Bisulfite-treated DNA was quantified using the quantitative real-time PCR QC1 methylight assay (4).

PCR amplification

Each locus was amplified in 20 µL PCR reactions in a Mastercycler Pro S (Eppendorf) using 20 ng of bisulfite-converted DNA as a template. The PCR mix included 1 X HotStar Taq DNA polymerase buffer, 1.8 mM of additional $MgCl_2$, 200 µM of each dNTP, 200 nM of each primer including one with a biotinylated tag (Table S4), and 2 U of HotStar Taq DNA polymerase. Cycling conditions included an initial denaturation step performed for 10 min at 95°C, followed by 50 cycles of 30 sec denaturation at 95°C, 30 sec annealing at 58°C and 30 sec elongation at 72°C. The final step included 5 min elongation at 72°C.

DNA methylation analysis by pyrosequencing

10 µl of each PCR product were purified and prepared for pyrosequencing according to a previously described protocol (5, 6). Pyrosequencing reactions were performed using the PyroMark Gold SQA Q96 Kit (Qiagen) on a PyroMark Q96 MD (Qiagen). DNA methylation analysis was performed using the PyroMark CpG software (Qiagen).

Predictive models and statistical analyses

All age-prediction model developments and statistical analyses were performed using R (https://www.r-project.org/). All graphical representations were performed using R or GraphPad Prism 10 (GraphPad Software, LLC). Age-prediction models were developed using DNA methylation values from the six selected CpG sites, applying elastic net regression (ENR) (7), gradient boosting regressor (GBR) (8), support vector machines (9), and Klemera-Doubal regression (KDR) (10). ENR is the gold standard regression approach used in numerous e-clocks (11, 12), the GBR and $SVM_{l/r}$, are two machine-learning-based classification approaches performing well in our previously developed clocks (13), while

KDR is used to develop estimators of biological age but requires chronological age as input (10, 14). The full DNA methylation dataset (n = 351) was randomly divided into training and testing sets using an 80/20 split (n = 283 and n = 68, respectively). Models were fitted on the training set and age predictions were evaluated on the testing set. For elastic net regression, the regularization parameter λ was optimized using ten-fold cross-validation, while the mixing parameter α was varied between 0 (ridge regression) and 1 (lasso regression). For the support vector machines, we tested polynomial (SVM_p), linear (SVM_l), and radial (SVM_r) kernels, but only results from the latter two kernel types are presented due to their improved performances. For the GBR, we optimized model performance by varying the number of boosting iterations (decision trees). For Klemera-Doubal regression, the model requires the chronological age of the samples as an input, as the method was specifically developed to predict biological age. For three CpGs, DNA methylation values (x) were also transformed to improve their linear relationship with age: $(x+1)^2$ for *ELOVL2* and $log_2\,(x\,+1)$ for *CCDC102B* and *EDARADD*. The transformed values, along with the untransformed DNA methylation values of the other three CpGs, were standardized (i.e., centered and scaled based on the training set) — except for GBR — and used to construct the age-prediction models, following the same procedure described above. For each age-prediction model, correlation was assessed using Pearson's *r*, and prediction accuracy was evaluated using the coefficient of determination R^2, the root mean square error (RMSE) and the mean absolute error (MAE).

Data and Code availability

All the data and source code are available in the Supplementary Materials.

Supplementary Figures

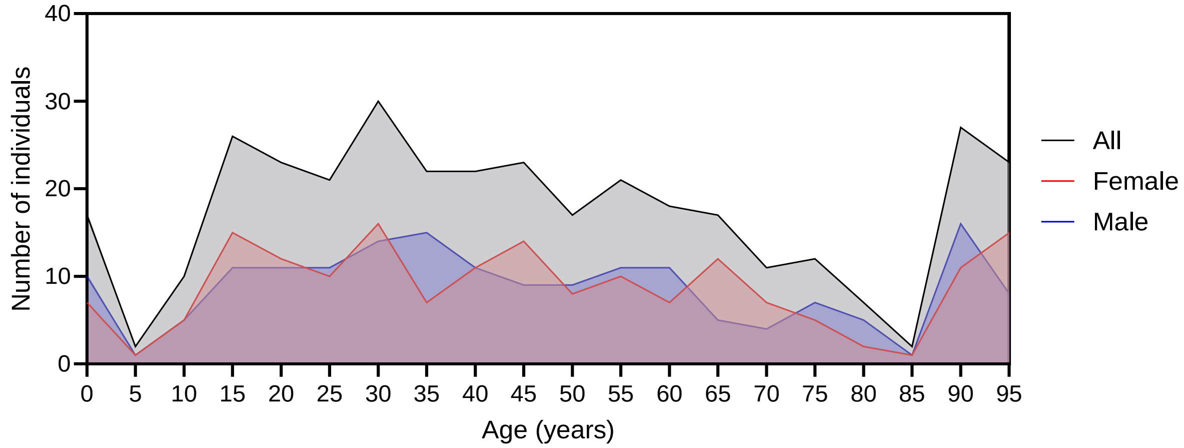

Figure S1: Age and sex distribution of the 351 DNA samples used in the study, including 176 female samples and 175 male samples.

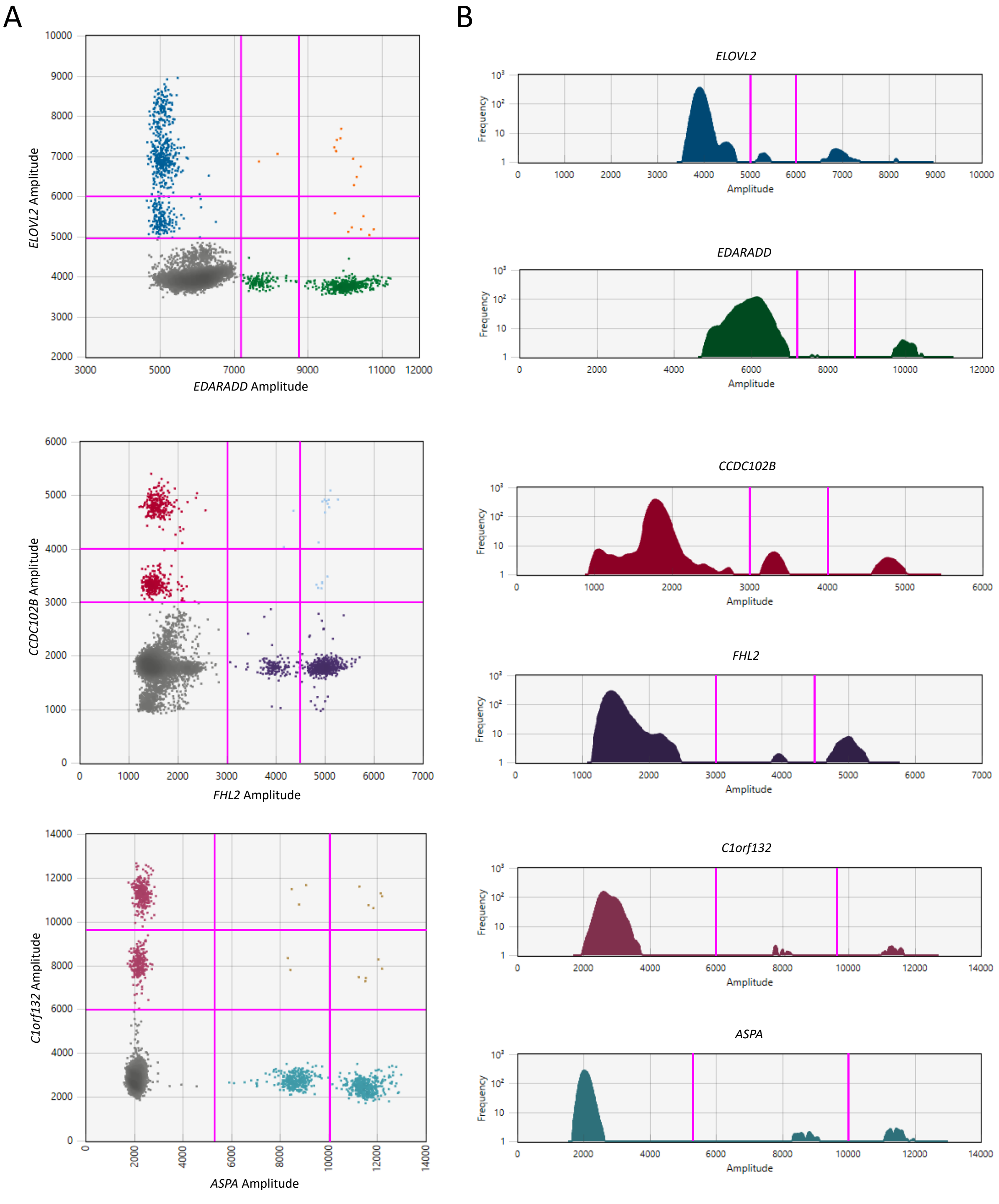

Figure S2: Double-threshold setting strategies for DNA methylation analysis by ddPCR using the 12 o'clock assay. The thresholds were set up for the six targeted CpGs using (**A**) 2D amplitude plots and (**B**) the corresponding 1D amplitude plots on a log scale. Two thresholds were manually set for each CpG in each sample: a lower threshold to count all positive droplets, and a higher threshold to count either the methylated droplets (*CCDC102B* and *ELOVL2*) or the unmethylated droplets (*ASPA*, *C1orf132*, *EDARADD* and *FHL2*) in the upper clusters. The example plots are from two whole blood DNA samples. For each CpG the higher threshold is set equidistant from the top of the lower and upper positive clusters. For *ELOVL2*, three distinct positive clusters were observed in some blood samples, which were not observed in DNA methylation standards. This additional cluster was attributed to variations in probe hybridization on target sequences containing either one or two methylated CpGs. The two upper clusters were thus grouped together for droplet analysis and DNA methylation quantification. For *CCDC102B*, some negative droplets form distinct clusters due to a color compensation issue that cannot be corrected manually. The lower threshold should be set to avoid these droplets.

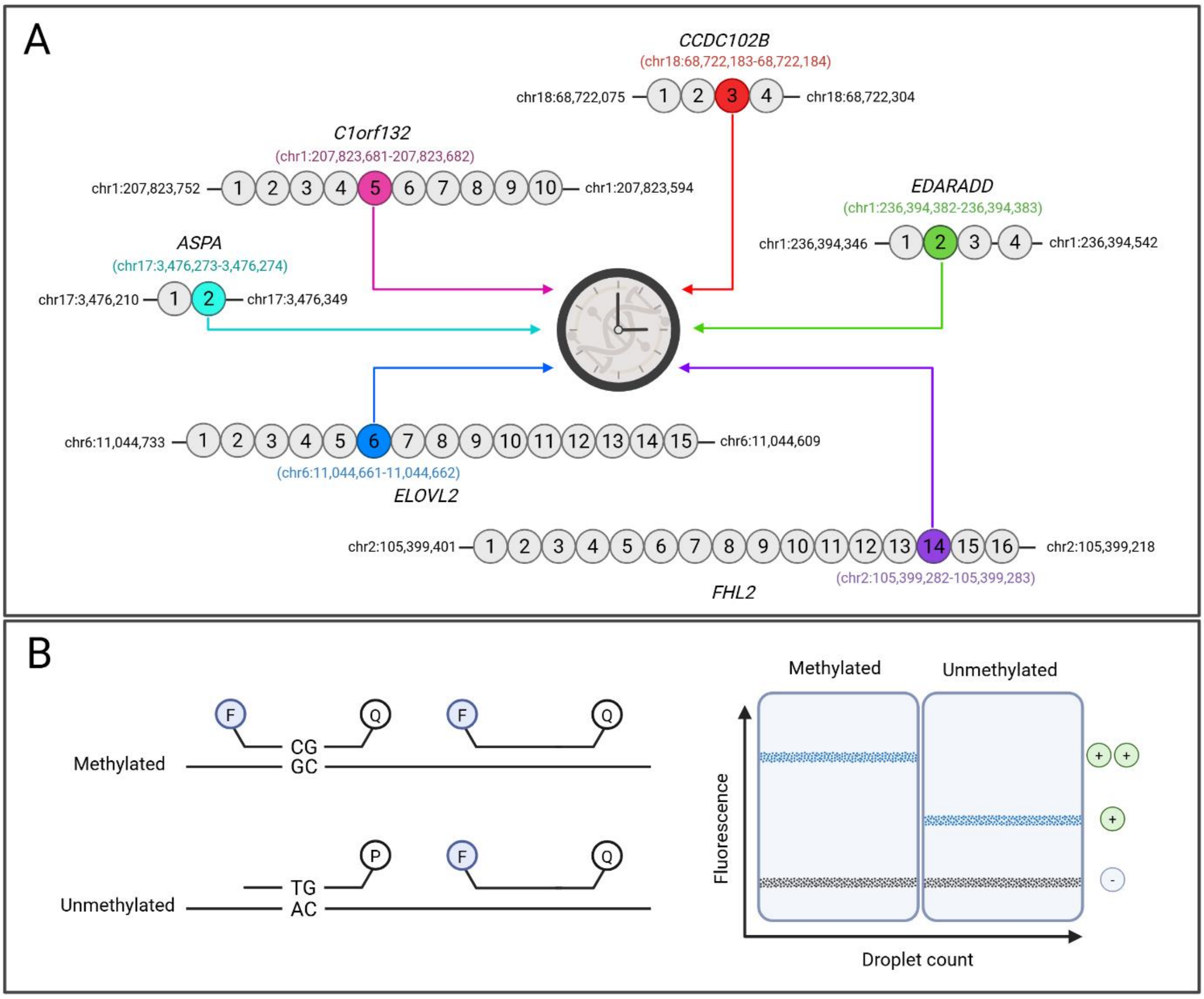

Figure S3: Overview of the 12 o'clock assay. **A.** Representation of the six CpG sites used in the ddPCR assay for the development of the epigenetic clocks. The CpG sites from each PCR amplicon are numbered according to the transcription direction of the associated genes, and those used for the epigenetic clocks are colored. The exact genomic locations of the CpGs analyzed by ddPCR are indicated in brackets according to the hg38 build. **B.** Principle of our ddPCR assays using two fluorescent and one invisible hydrolysis probes to detect the methylated and unmethylated CpG of the same locus. F: fluorescent dye. Q: quencher. P: phosphate.

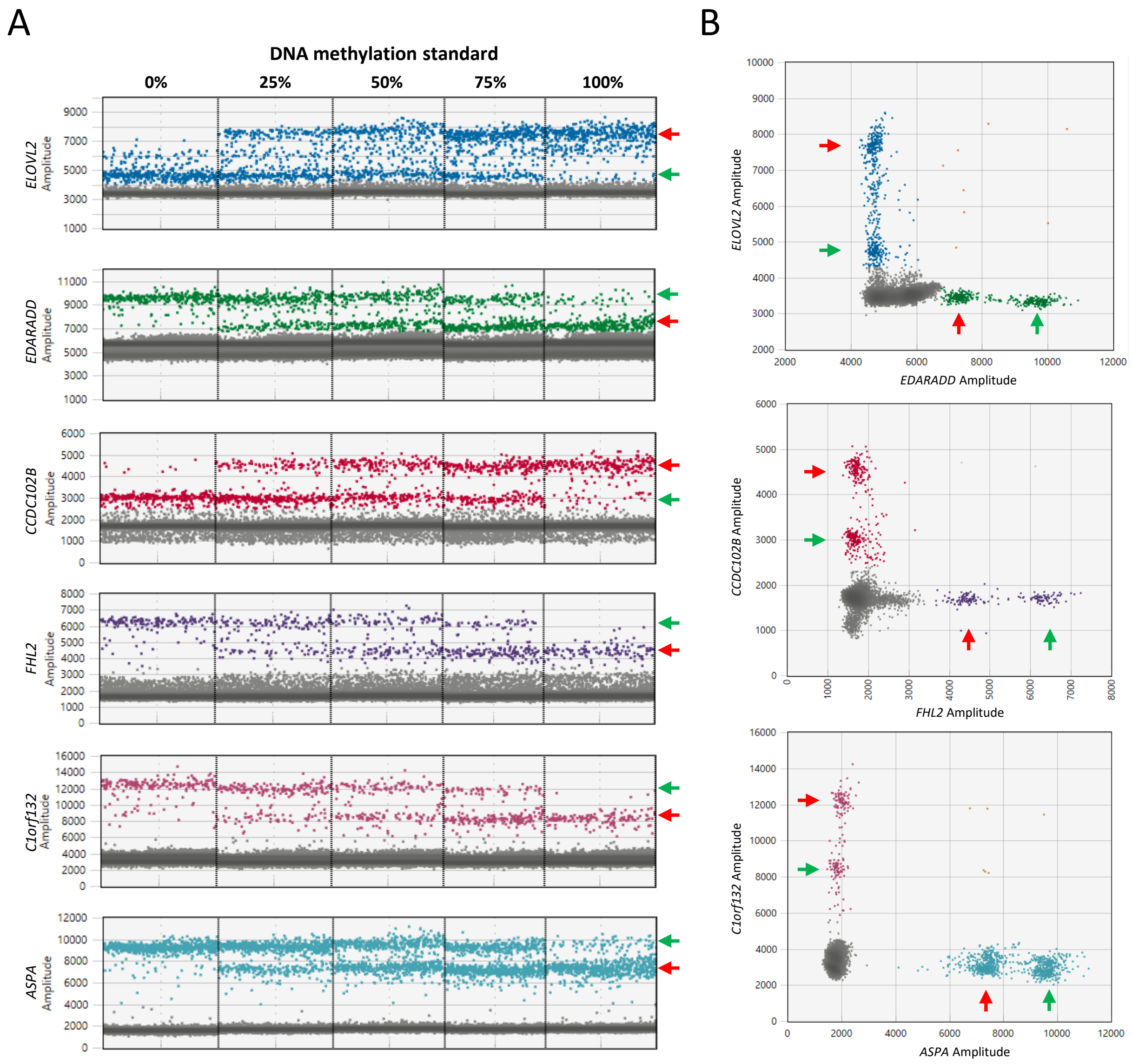

Figure S4: Examples of ddPCR results obtained using the 12 o'clock assay on DNA methylation standards at the six CpG sites. **A.** Representative 1D amplitude plots obtained with 0%, 25%, 50%, 75% and 100% DNA methylation standards. **B.** Representative 2D amplitude plots obtained with 50% DNA methylation standard. 10 ng of DNA methylation standards were used per ddPCR and PCR reaction. Red and green arrows indicate the methylated and unmethylated alleles.

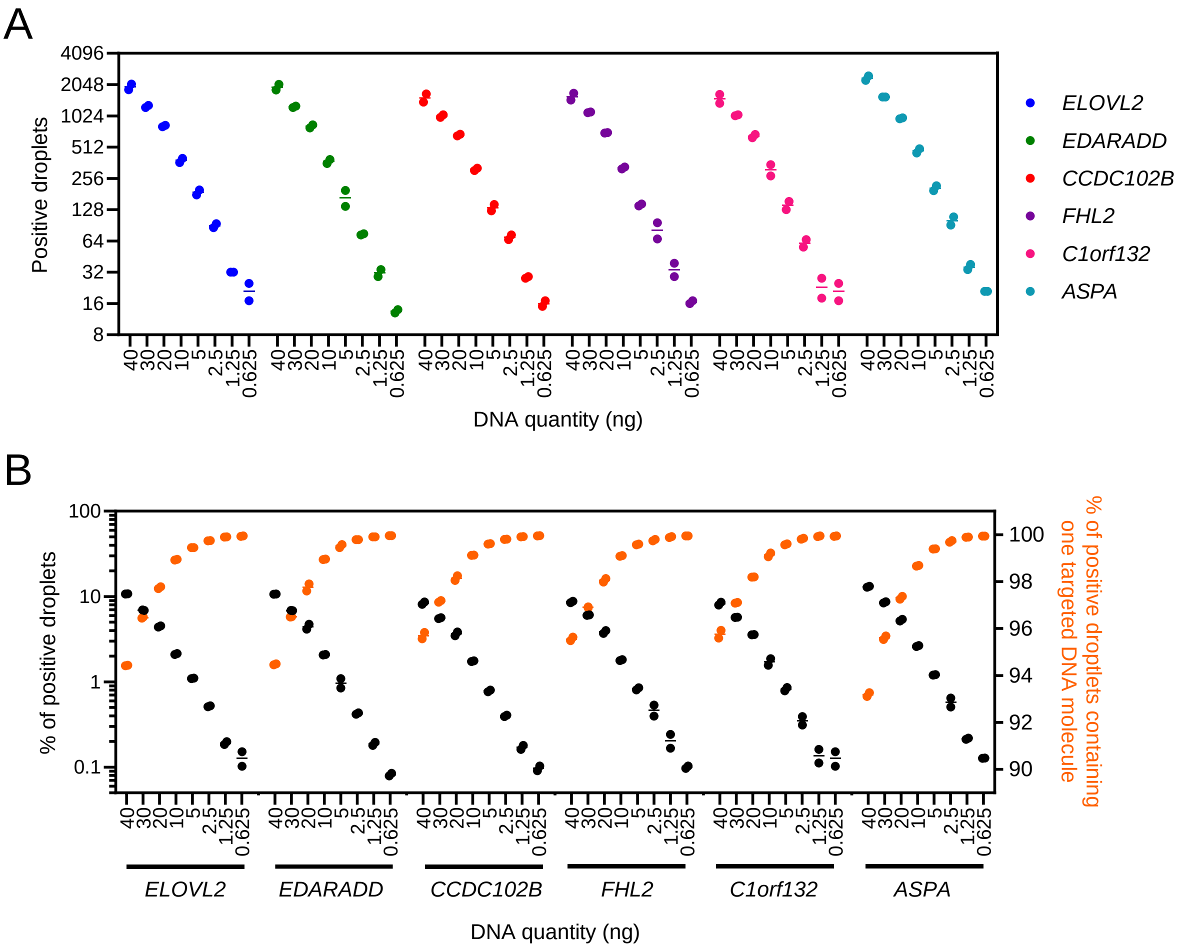

Figure S5: Evaluation of ddPCR experimental metrics using the 12 o'clock assay with decreasing amounts of bisulfite-converted whole blood DNA (Promega). **A**. Number of positive droplets obtained for the six CpG sites. **B**. Percentages of positive droplets (black dots) and of those containing a single targeted molecule at partitioning, estimated from the Poisson distribution (orange dots), for the six CpG sites. Each experiment was performed in duplicate. Individual values are shown as dots, and the mean of the duplicates is represented as a bar.

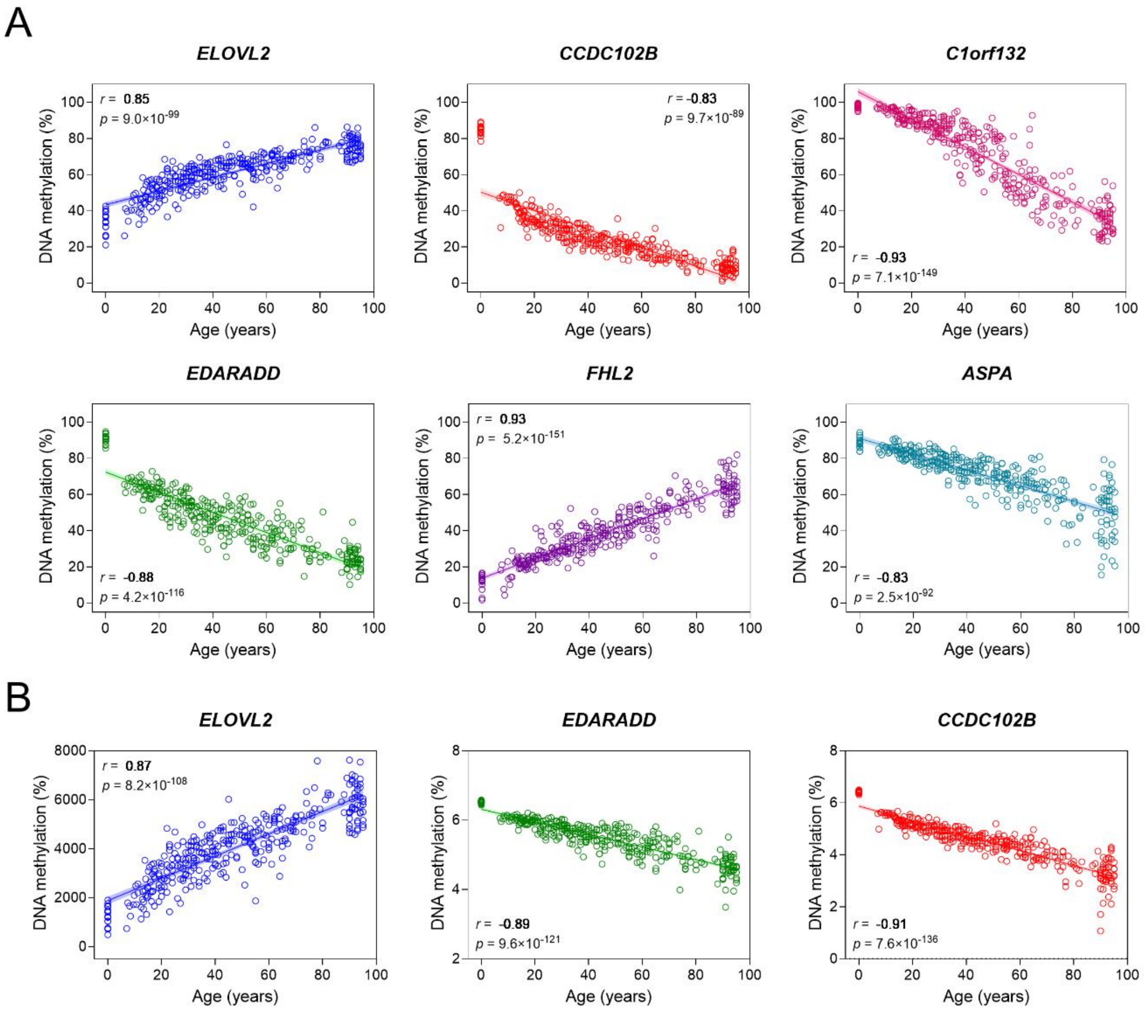

Figure S6: Correlation analysis of DNA methylation and age for the six CpG sites using the 351 DNA samples. **A**. Correlation between DNA methylation levels directly measured by ddPCR and age. **B**. Correlation between transformed DNA methylation levels of *ELOVL2*, *EDARADD* and *CCDC102B* and age. Pearson's *r* coefficient and associated *p*-value are indicated in each plot, along with the 95% confidence interval of the regression line. Transformations included squaring the DNA methylation values of *ELOVL2* after adding 1 and applying $\log_2$ transformation to DNA methylation values of *EDARADD* and *CCDC102*, also after adding 1. The large differences in DNA methylation levels observed between cord blood and the youngest children for *EDARADD* and *CCDC102B* suggest a more pronounced methylation change at these loci during early life than during adulthood, consistent with previous reports (15-17). Moreover, these differences may also be partly explained by the marked differences in cell composition between cord blood and blood from older individuals (18, 19).

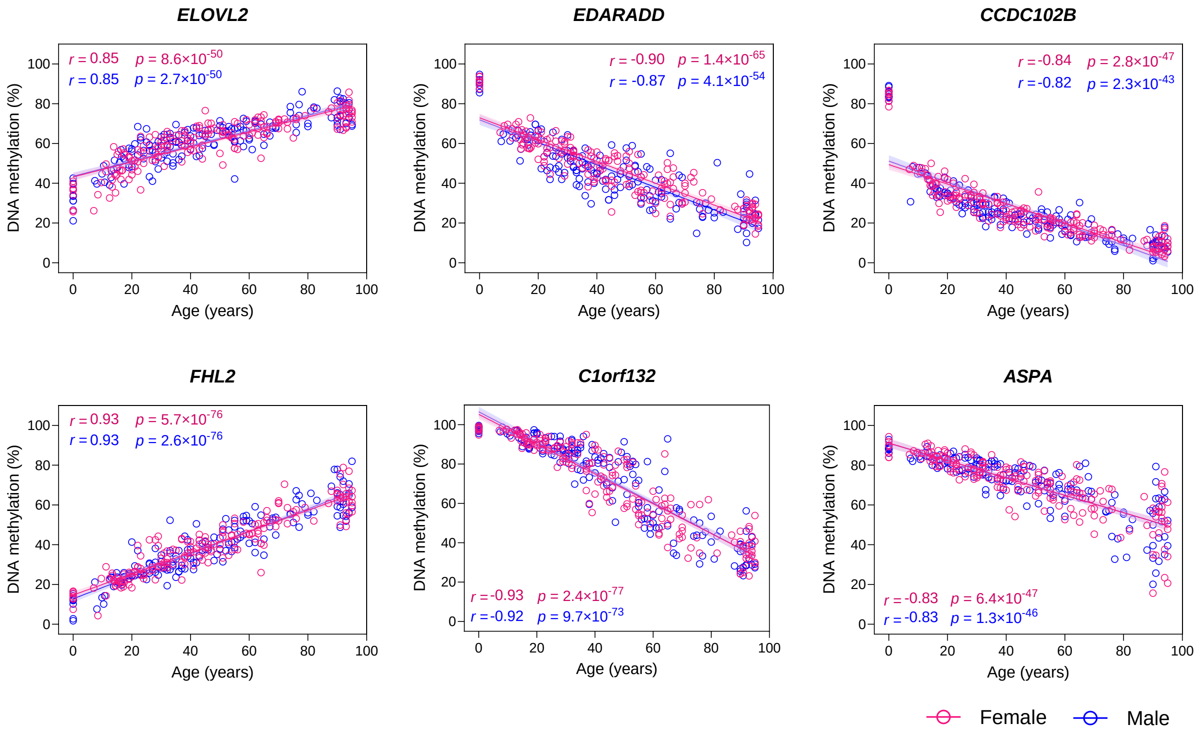

Figure S7: Correlation analysis of DNA methylation and age according to sex for the six CpG sites using the 351 DNA samples (n = 351, 175 male samples and 175 female samples). Pearson's *r* coefficients and associated *p*-values are indicated in each plot, along with the 95% confidence interval of the regression line.

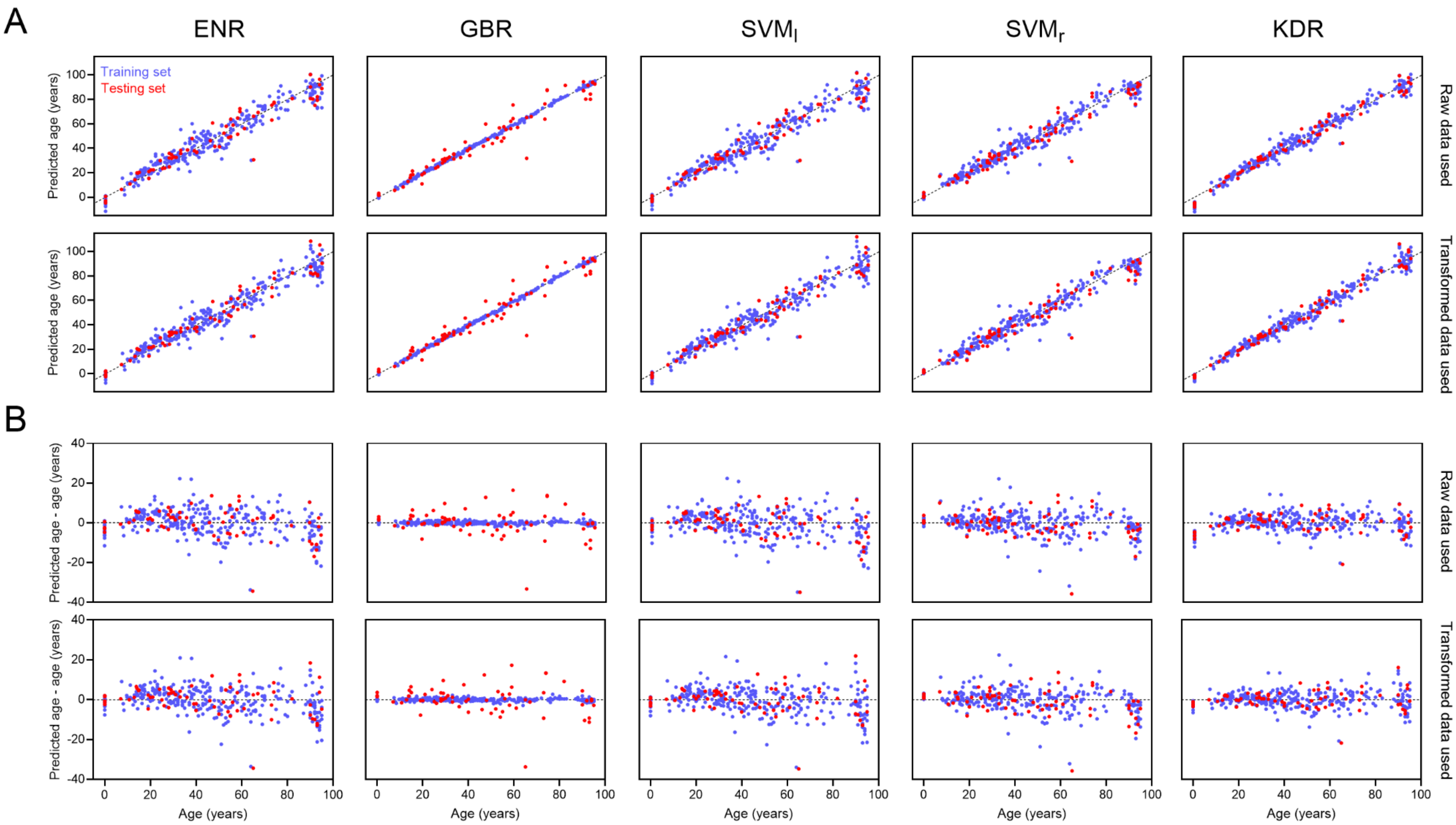

Figure S8: Comparison of the different epigenetic clock predictions using DNA methylation of the six CpGs and five different models (ELN, GBR, SVM_l, SVM_r and KDR). **A**. Scatterplots of predicted ages and chronological ages. **B**. Differences between predicted ages and chronological ages. For the transformed data, DNA methylation of *ELOVL2*, *EDARADD* and *CCDC102B* were first transformed, and methylation levels of all six CpG were then standardized prior to model construction (see Methods).

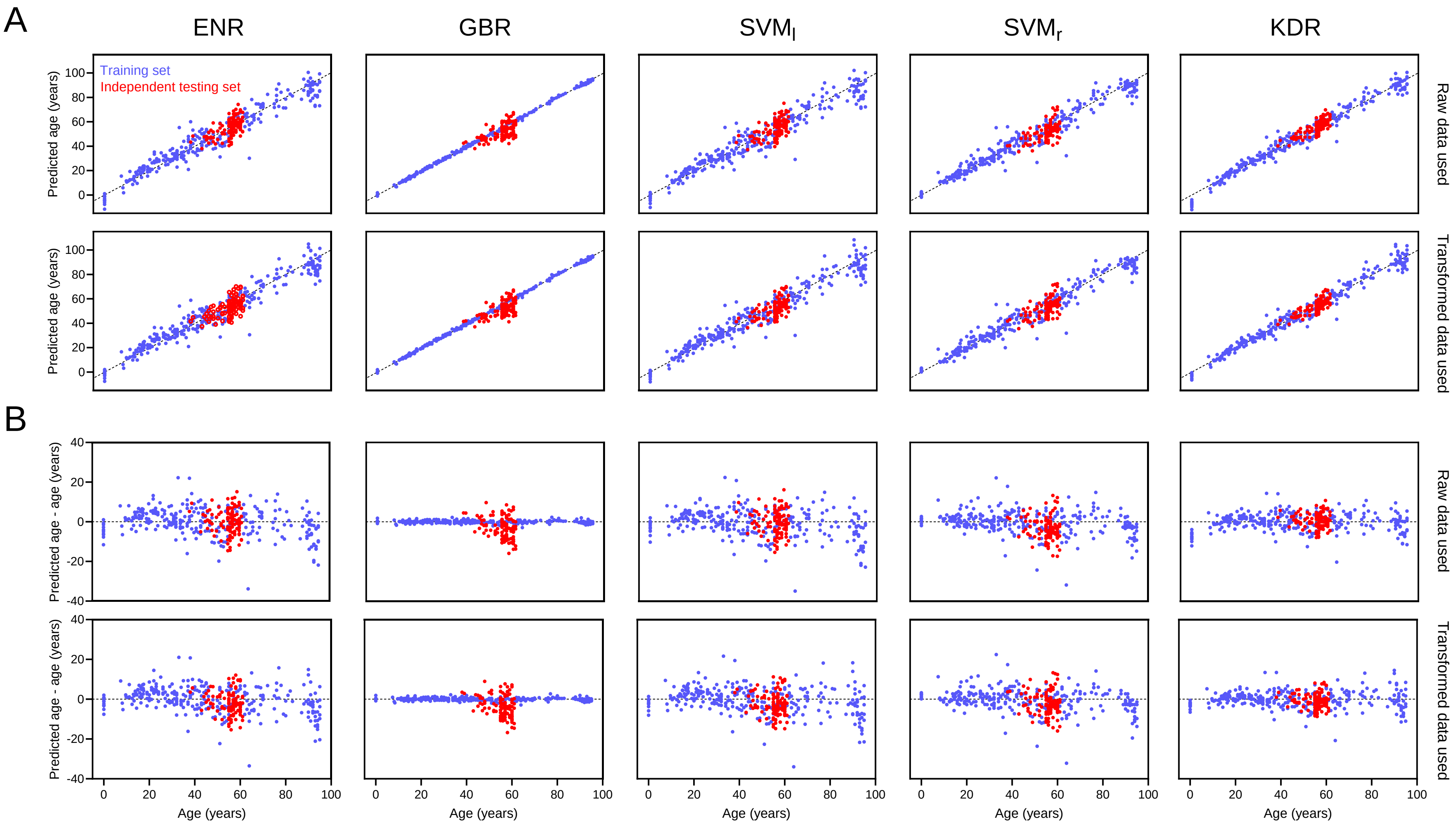

Figure S9: Comparison of the different epigenetic clock predictions from an independent testing set (SU.VI.MAX cohort, n = 116) using DNA methylation of the six CpGs and five different models (ELN, GBR, SVM$_l$, SVM$_r$ and KDR). **A**. Scatterplots of predicted ages and chronological ages. **B**. Differences between predicted ages and chronological ages. For the transformed data, DNA methylation of *ELOVL2*, *EDARADD* and *CCDC102B* were first transformed, and methylation levels of all six CpG were then standardized prior to model construction (see Methods).

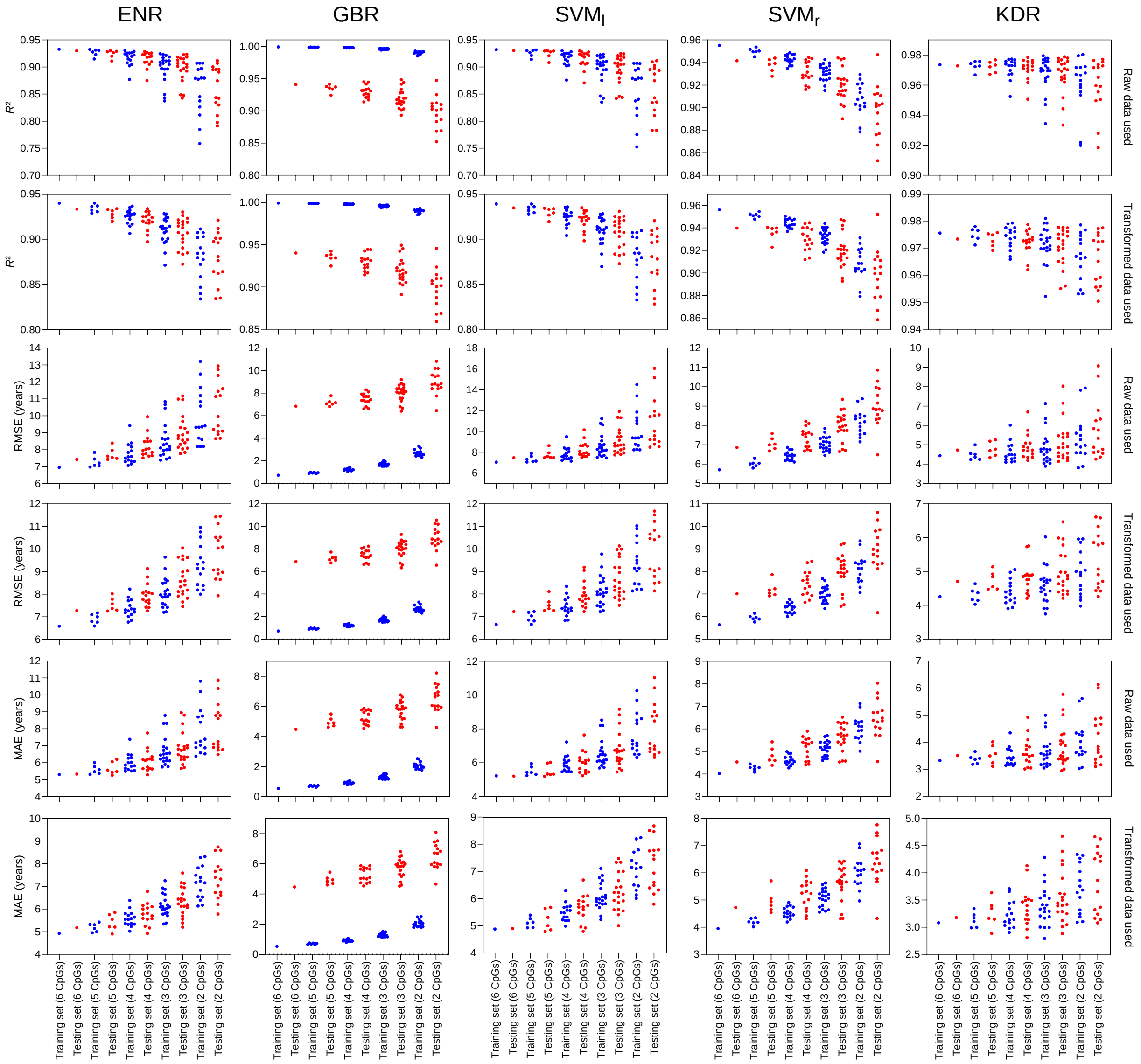

Figure S10: Comparison of age prediction performance (R^2, RMSE, and MAE) for epigenetic clocks developed using all combinations of 2 to 6 CpG sites among the six targeted genes. Performance metrics were calculated separately for training and testing set samples. The six statistical models (ELN, GBR, SVM_l, SVM_r, and KDR) used to build the epigenetic clocks are grouped according to the type of DNA methylation data used (raw or transformed). For transformed data, only the DNA methylation values of *ELOVL2*, *EDARADD*, and *CCDC102B* were transformed, while methylation levels of all six CpGs were standardized prior to model construction (see Methods).

Supplementary Tables

Table S1: Advantages and limitations of different DNA methylation analysis methods used with epigenetic clocks

DNA methylation analysis methods	Throughtput	Advantages	Limitations
Pyrosequencing (13, 20, 21)	Low	- Good DNA methylation quantification resolution (±5–7%)<br>- Multiple CpGs per amplicon (up to 140 nt) can be analyzed, allowing evaluation of different CpGs per locus<br>- Flexible assay design; relatively insensitive to sequence context<br>- Easy to handle and cost-effective	- Multiplexing not feasible<br>- Quantification sensitive to PCR biases (low to high depending on assay)<br>- Sensitive to batch effects and interlaboratory variations<br>- Requires PCR followed by sequencing<br>- Limited support for sophisticated analyses
SNaPshot™ (22-26)	Low	- Multiplexing possible for PCR, single-base extension, and capillary electrophoresis fragment analysis<br>- Multiple CpGs per amplicon (up to 10) can be analyzed, allowing evaluation of different CpGs per locus<br>- Easy to handle and cost-effective	- Requires multiple post-PCR steps (single-base extension and capillary electrophoresis)<br>- Lower DNA methylation quantification resolution, accuracy, and reproducibility than pyrosequencing, amplicon sequencing, and EpiTYPER©<br>- Quantification sensitive to PCR biases (low to high depending on assay)<br>- Sensitive to batch effects and interlaboratory variations<br>- Limited support for sophisticated analyses
EpiTYPER© (21, 22, 27-29)	Low	- Good DNA methylation quantification resolution (comparable to pyrosequencing)<br>- Multiple CpGs per amplicon can be analyzed, allowing evaluation of different CpGs per locus<br>- Can analyze long DNA sequences (up to 600 nt) in a single experiment<br>- High sample throughput (up to 384 samples per run)<br>- Easy to handle and cost-effective	- Requires multiple post-PCR steps (*in vitro* transcription and mass spectrometry)<br>- Multiplex PCR not performed<br>- Some closely spaced CpGs cannot be analyzed separately and are grouped and are reported as average DNA methylation<br>- Quantification sensitive to PCR biases (low to high depending on assay)<br>- Sensitive to batch effects and interlaboratory variations<br>- Limited support for sophisticated analyses
ddPCR (26, 30-36)	Low	- Enables absolute DNA methylation quantification<br>- Multiplexing feasible<br>- DNA methylation detected at endpoint after PCR (no additional post-PCR steps required)<br>- Direct quantification of amplified molecules (allows control for stochastic effects)<br>- Reduces batch effects and interlaboratory variation<br>- Easy to handle and cost-effective	- Limited number of loci per assay<br>- Certain CpGs in complex sequence context cannot be targeted<br>- Cannot easily analyze two closely spaced CpGs in the same assay<br>- Fluorescent probes can be expensive, especially during optimization<br>- Limited support for sophisticated analyses
Amplicon (short-reads) sequencing	Low to mid	- Good DNA methylation quantification resolution (comparable to pyrosequencing)<br>- Samples can be highly multiplexed in the same NGS run	- Multiplex PCR on bisulfite-treated DNA is challenging, usually requiring simplex PCR amplicons pooled before sequencing<br>- Quantification sensitive to PCR biases (low to high depending on assay)

(22, 23, 26, 37, 38)		- Multiple CpGs per amplicon can be analyzed, allowing evaluation of different CpGs per locus<br>- Assay design feasible for specific CpGs, less sensitive to sequence context<br>- Can interrogate larger panels of CpGs compared to low-throughput methods<br>- Provides a compromise between low- and high-throughput approaches	- Sensitive to batch effects and interlaboratory variation<br>- Requires PCR followed by sequencing<br>- Requires bioinformatics analysis<br>- Limited support for sophisticated analyses
Illumina Infinium Methylation BeadChips (27K, 450K and EPIC/~850K) (21, 39-42)	High	- Can interrogate hundreds of thousands of CpGs in a single experiment (ideal for screening age-related biomarkers)<br>- Array design allows some compatibility across versions<br>- Supports sophisticated bioinformatic analyses (cell type adjustment, epigenetic clocks, probes compatible with human-related species)<br>- Large number of public datasets available	- Requires relatively high DNA input (500–1,000 ng)<br>- Low quantitative resolution (10–15%)<br>- Only CpGs included on the array can be interrogated<br>- Certain CpGs in complex sequence contexts cannot be targeted, even in custom design<br>- Very sensitive to batch effects and interlaboratory variation (array version, missing data, preprocessing/normalization)<br>- Requires bioinformatics analysis for newly generated data
RRBS (Reduced Representation Bisulfite Sequencing) (43)	High	- Can interrogate hundreds of thousands of CpGs in a single experiment (ideal for screening age-related biomarkers)<br>- Samples can be highly multiplexed in the same NGS run<br>- Cost-effective NGS-based method<br>- Requires less DNA than epigenotyping microarrays<br>- Supports sophisticated bioinformatic analyses (e.g., cell type adjustment)	- Low quantitative resolution due to low and variable coverage across CpG sites<br>- Loss of data on CpGs depending on coverage thresholds<br>- Less standardized than epigenotyping microarrays<br>- Highly sensitive to batch effects and interlaboratory variation (coverage and quality differences across datasets)<br>- Requires bioinformatics analysis of newly generated data

Table S2: Descriptive statistics of the 351 blood extracted DNA samples used in our study.

Cohort	IRSL (n = 17)	STANISLAS (n = 40)	EFS (n = 196)	CEPH Aging (n = 98)
Age (years),<br>Mean ± SD (range)	0.0 ± 0.0 (0-0)	13.7 ± 3 (7-17.5)	39.4 ± 13.0 (19-66)	81.6 ± 12.1 (55-95)
Females, n (%)	7 (41.18%)	22 (55%)	96 (48.98%)	51 (52.04%)

Table S3: List of all primers and probes used in the 12 o'clock multiplex ddPCR assay

Gene name	Primers and probes	Sequences[1, 2]	Final concentrations in ddPCR reaction (nM)[3]	Amplicon length (pb)
	Forward primer	GGYGATTTGTAGGTTTAGTYGG	900	
	Reverse primer	CCTACACRATACTACTTCTCCC	900	
ELOVL2	Universal probe	FAM-TTGTT+TAGAT+YGGTAGT+YGTTGTTG-BHQ1	250	125
	Methylated allele probe	FAM-CTAAAACTCC**G**TAAACRTTAAACC-BHQ1	300	
	Unmethylated allele probe	CTAAAACTC+C**A**TAAA+CRTTAAACC-PHOSPHATE	300	
	Forward primer	TTTGAGAAATTTAGGAAGATAGTAAATG	900	
	Reverse primer	ATTTAACTCTAACCAAACAACCAAC	900	
EDARADD	Universal probe	HEX-AACA+CTAAAACT+CCTAATCAC-BHQ1	300	197
	Methylated allele probe	CAT+CTTCTC**G**CAAT+CTATT-PHOSPHATE	250	
	Unmethylated allele probe	HEX-CAT+CTTCT+C**A**CAAT+CTATT-BHQ1	250	
	Forward primer	TAAGGGAAGAGTGGGGAAATTG	300	
	Reverse primer	ACCTTACCCTAATAAAACTTTATTCA	300	
CCDC102B	Universal probe	CY5-ATTAT+CT+CTAA+CTTTAAA+CGCTA-BHQ3	85	230
	Methylated allele probe	CY5-ACACTCATCCC**G**CATCCTT-BHQ3	85	
	Unmethylated allele probe	ACACTCATC+CC**A**CATCCTT-PHOSPHATE	85	
	Forward primer	GTATTTTTTGTTTGTTAGGGTTTT	300	
	Reverse primer	CTCCTAAAACCAAACAAAAATCC	300	
FHL2	Universal probe	CY5.5-TAAA+CAAACCCCTA+CCACC-BBQ3	85	170
	Methylated allele probe	ACRCTCCC**G**ATAACTACTATACTCC-PHOSPHATE	85	
	Unmethylated allele probe	CY5.5-ACRCTCCC**A**ATAACTACTATACTCCCA-BHQ3	85	
	Forward primer	GTTTGATGGGATTAAATTTTGGAATAT	650	
	Reverse primer	CCCTAATCCCAACAAATACATAC	650	
C1orf132	Universal probe	ROX-CCAT+CAAATC+CTAAAA+CCRTC-BHQ2	165	159
	Methylated allele probe	CCRCCTAC**G**CAAACCCAA-PHOSPHATE	165	
	Unmethylated allele probe	ROX-CCRC+CTAC**A**+CAAACCCAA-BHQ2	165	
	Forward primer	TGGAGGAATTTATGGGAATGA	650	
	Reverse primer	ATCTAATACACTTCTTCACTACTCT	650	
ASPA	Universal probe	ATTO590-TTTAC+CTCCAAC+CCTATT+CTC-BHQ2	165	140
	Methylated allele probe	AAATCT+CAAC**+G**CCA+TTCTCTAAC-PHOSPHATE	165	
	Unmethylated allele probe	ATTO590-AAATCT+CAAC**+A**CCA+TTCTCTAAC-BHQ2	165	

[1] The methylated and unmethylated cytosines detected by each probe after bisulfite conversion are shown in bold in the sequence.

[2] Some probes incorporated LNA bases to increase melting temperature while keeping sequences short, and degenerate nucleotides when additional CpGs were present in the probe region. The invisible probe was essential for allele-specific detection, introducing probe competition that prevents false-positive signals.

[3] Our optimization procedure involved testing each locus individually on DNA methylation standards, starting from the Bio-Rad recommended concentrations (900 nM primers, 250 nM probes) and decreasing by factors of 1.5, 2, 3, and 4. For each locus, the lowest concentrations that maintained good cluster separability were used as the starting point for multiplex experiments, which were then fine-tuned with minor adjustments to reach the final optimized values.

Table S4: List of all PCR and pyrosequencing primers used

Gene name	Primers	Sequences	Annealing temperature (°C)	Sequence to analyze by pyrosequencing[1]	Amplicon length (pb)
	Forward PCR primer	Biotin-GGYGATTTGTAGGTTTAGTYGG	58		
ELOVL2	Reverse PCR primer	CCTACACRATACTACTTCTCCC			125
	Pyrosequencing primer	ACAACRAATAAATATTCCTAAAACT		C**CR**TAAACRTTAAACCRCCRCRCRAAACCRAC	
	Forward PCR primer	TTTGAGAAATTTAGGAAGATAGTAAATG	58		
EDARADD	Reverse PCR primer	Biotin-ATTTAACTCTAACCAAACAACCAAC			197
	Pyrosequencing primer	GGAGTTTGTTATGGAAGAAGTAATAGATTG		**YG**AGAAGATGTTYGTTGG	
	Forward PCR primer	Biotin-TAAGGGAAGAGTGGGGAAATTG	58		
CCDC102B	Reverse PCR primer	ACCTTACCCTAATAAAACTTTATTCA			230
	Pyrosequencing primer	AAAATATTTACACTCATCCC		**R**CATCCTTTTTTAACTAC	
	Forward PCR primer	GTATTTTTTGTTTGTTAGGGTTTT	58		
FHL2	Reverse PCR primer	Biotin-CTCCTAAAACCAAACAAAAATCC			170
	Pyrosequencing primer	GGTTTTGGGAGTATAGT		AGTTAT**YG**GGAGYGTYGTTTTYGGYGTGGG	
	Forward PCR primer	GTTTGATGGGATTAAATTTTGGAATAT	58		
C1orf132	Reverse PCR primer	Biotin-CCCTAATCCCAACAAATACATAC			159
	Pyrosequencing primer	GGATTAAGATTTGGGTTTG		**YG**TAGGYGGYGATGGGTGG	
	Forward PCR primer	Biotin-TGGAGGAATTTATGGGAATGA	58		
ASPA	Reverse PCR primer	ATCTAATACACTTCTTCACTACTCT			140
	Pyrosequencing primer	CCCTATTCTCTAAATCTCAA		**CR**CCATTCTCTAACCAATAC	

[1] The CpG sites analyzed using both ddPCR and pyrosequencing are highlighted in bold and underlined.

Table S5: Performances of four pyrosequencing-based e-clocks using the independent testing set (n = 116)

**Age-prediction model**[1]	**Biomarkers included in the models**	***r***	**R^2**	**MAE**	**RMSE**
Thong clock	*ELOVL2*, *KLF14*, *TRIM59*	0.7	0.490	5.43	6.67
Bekaert clock	*ASPA*, *ELOVL2*, *EDARADD*, *PDE4C*	0.6457	0.417	6.24	7.40
Garali MQR clock	*ELOVL2*	0.5186	0.269	9.88	11.25
Garali GBR clock		0.4821	0.232	10.17	11.59

[1] The performances were calculated using our previously published data (3).

Table S6: Age prediction performances of the different epigenetic clocks based on different CpG combinations using raw DNA methylation data.

CpG combination	CpG nb.	ENR						GBR						SVM_l						SVM_r						KDR					
		Training set			Testing set			Training set			Testing set			Training set			Testing set			Training set			Testing set			Training set			Testing set		
		MAE	RMSE	R^2	MAE	RMSE	R^2	MAE	RMSE	R^2	MAE	RMSE	R^2	MAE	RMSE	R^2	MAE	RMSE	R^2	MAE	RMSE	R^2	MAE	RMSE	R^2	MAE	RMSE	R^2	MAE	RMSE	R^2
ELOVL2+EDARADD	2	8.75	10.84	0.84	8.95	11.19	0.84	1.98	2.59	0.99	6.02	8.85	0.90	8.53	11.02	0.84	9.02	11.58	0.84	5.92	8.19	0.91	6.72	9.54	0.89	5.80	4.34	0.96	6.24	4.89	0.96
ELOVL2+CCDC102B	2	10.81	13.21	0.76	10.38	12.74	0.80	1.87	2.55	0.99	5.77	7.75	0.93	10.26	14.49	0.75	11.03	16.04	0.78	5.62	7.74	0.92	6.74	8.82	0.90	7.93	5.62	0.92	8.55	6.13	0.93
ELOVL2+FHL2	2	7.03	9.35	0.88	7.10	9.20	0.90	2.13	2.81	0.99	7.25	9.45	0.89	7.01	9.37	0.88	6.98	9.20	0.90	5.94	8.39	0.90	6.48	8.84	0.90	5.01	3.80	0.97	4.76	3.73	0.97
ELOVL2+C1orf132	2	6.53	8.19	0.91	6.77	9.11	0.90	2.06	2.70	0.99	6.63	8.77	0.90	6.50	8.23	0.91	6.65	9.22	0.89	6.11	7.92	0.91	6.36	8.76	0.90	3.88	3.07	0.98	4.36	3.10	0.98
ELOVL2+ASPA	2	8.70	11.20	0.83	8.79	11.60	0.83	2.52	3.29	0.99	7.54	10.19	0.87	8.61	11.30	0.82	8.75	11.94	0.82	6.98	9.25	0.88	7.60	9.90	0.88	5.60	4.26	0.96	5.76	4.33	0.96
EDARADD+CCDC102B	2	10.19	12.47	0.78	10.87	12.94	0.79	1.81	2.42	0.99	5.81	8.50	0.91	9.70	13.40	0.78	10.43	15.15	0.78	6.23	8.49	0.90	6.38	8.86	0.90	7.82	5.52	0.92	9.07	6.01	0.92
EDARADD+FHL2	2	6.88	8.69	0.90	6.90	8.90	0.90	1.96	2.51	0.99	6.03	8.39	0.91	6.74	8.75	0.89	6.61	8.77	0.90	5.59	7.57	0.92	5.70	8.32	0.91	4.54	3.54	0.97	4.60	3.46	0.97
EDARADD+C1orf132	2	6.90	8.62	0.90	7.25	9.96	0.87	2.14	2.67	0.99	6.96	9.61	0.88	6.86	8.66	0.90	7.14	10.02	0.87	6.33	8.12	0.91	6.81	9.96	0.88	4.59	3.67	0.97	5.34	3.84	0.97
EDARADD+ASPA	2	8.39	10.58	0.85	8.78	11.13	0.84	2.50	3.14	0.99	8.23	10.82	0.85	8.32	10.75	0.84	8.78	11.41	0.84	7.13	9.38	0.88	8.03	10.86	0.85	5.47	4.29	0.96	5.83	4.61	0.96
CCDC102B+FHL2	2	7.27	9.33	0.88	7.08	9.08	0.90	1.81	2.45	0.99	4.60	6.45	0.95	7.17	9.37	0.88	6.85	9.01	0.90	5.02	7.15	0.93	4.55	6.48	0.95	4.88	3.62	0.97	4.87	3.62	0.97
CCDC102B+C1orf132	2	6.58	8.19	0.91	6.92	9.41	0.89	1.78	2.30	0.99	5.95	8.39	0.91	6.50	8.28	0.91	6.81	9.58	0.89	5.39	7.36	0.93	5.73	8.56	0.91	3.81	3.02	0.98	4.56	3.23	0.98
CCDC102B+ASPA	2	9.06	11.67	0.81	9.43	12.38	0.81	1.98	2.70	0.99	6.05	8.81	0.90	8.94	11.85	0.81	9.44	12.93	0.81	6.29	8.57	0.90	6.31	9.16	0.90	5.95	4.38	0.95	6.79	4.86	0.95
FHL2+C1orf132	2	6.39	8.19	0.91	6.49	8.65	0.91	1.83	2.50	0.99	6.85	9.54	0.89	6.31	8.22	0.91	6.32	8.52	0.91	5.67	7.54	0.92	6.04	8.35	0.91	4.58	3.55	0.97	4.64	3.37	0.97
FHL2+ASPA	2	7.18	9.33	0.88	6.74	8.67	0.91	2.17	2.77	0.99	6.74	8.70	0.91	7.10	9.37	0.88	6.49	8.48	0.91	6.13	8.33	0.90	6.09	8.12	0.92	4.97	3.64	0.97	4.26	3.16	0.98
C1orf132+ASPA	2	7.39	9.41	0.88	8.59	11.45	0.83	2.33	3.02	0.99	7.47	10.20	0.87	7.31	9.50	0.88	8.47	11.51	0.83	6.35	8.48	0.90	7.37	10.29	0.87	5.26	4.02	0.96	6.33	4.67	0.95
ELOVL2+EDARADD+CCDC102B	3	8.79	10.84	0.84	8.95	11.17	0.84	1.17	1.52	1.00	5.18	7.30	0.93	8.52	11.23	0.84	9.16	11.92	0.84	5.08	6.94	0.93	6.05	8.30	0.91	7.13	4.99	0.93	8.03	5.77	0.93
ELOVL2+EDARADD+FHL2	3	6.45	8.30	0.90	6.88	8.67	0.91	1.16	1.53	1.00	5.82	8.04	0.92	6.39	8.35	0.90	6.80	8.60	0.91	4.83	6.74	0.94	5.79	8.01	0.92	4.63	3.53	0.97	4.86	3.87	0.97
ELOVL2+EDARADD+C1orf132	3	6.09	7.67	0.92	6.37	8.59	0.91	1.19	1.58	1.00	5.87	8.09	0.92	6.08	7.69	0.92	6.31	8.68	0.91	5.21	6.98	0.93	5.97	8.19	0.92	4.05	3.19	0.98	4.59	3.47	0.97
ELOVL2+EDARADD+ASPA	3	7.37	9.42	0.88	7.76	9.95	0.87	1.34	1.74	1.00	6.62	8.76	0.90	7.25	9.52	0.88	7.68	10.13	0.87	5.42	7.39	0.92	6.25	8.85	0.90	5.08	3.84	0.97	5.46	4.28	0.96
ELOVL2+CCDC102B+FHL2	3	6.94	9.04	0.89	7.03	8.89	0.90	1.20	1.65	1.00	4.83	6.80	0.94	6.90	9.09	0.89	6.76	8.86	0.90	4.69	6.63	0.94	5.02	7.07	0.94	5.31	3.93	0.96	5.42	4.31	0.97
ELOVL2+CCDC102B+C1orf132	3	6.12	7.74	0.92	6.18	8.57	0.91	1.16	1.53	1.00	5.32	7.55	0.93	6.09	7.75	0.92	6.13	8.63	0.91	4.68	6.61	0.94	5.40	7.73	0.93	3.89	3.05	0.98	4.34	3.23	0.98
ELOVL2+CCDC102B+ASPA	3	8.32	10.66	0.84	8.29	10.96	0.85	1.39	1.91	1.00	5.81	7.95	0.92	8.20	10.76	0.84	8.34	11.30	0.85	5.39	7.39	0.92	6.29	8.44	0.91	6.13	4.57	0.95	6.63	5.01	0.95
ELOVL2+FHL2+C1orf132	3	5.75	7.45	0.92	5.72	7.85	0.92	1.28	1.70	1.00	6.42	8.72	0.90	5.73	7.50	0.92	5.56	7.78	0.92	5.07	6.87	0.93	5.65	7.74	0.92	4.15	3.18	0.98	4.18	3.01	0.98
ELOVL2+FHL2+ASPA	3	6.51	8.62	0.90	6.47	8.32	0.92	1.43	1.88	1.00	6.23	8.31	0.91	6.48	8.63	0.90	6.28	8.22	0.92	5.41	7.35	0.93	5.75	7.91	0.92	4.76	3.58	0.97	4.39	3.38	0.98
ELOVL2+C1orf132+ASPA	3	6.22	7.97	0.91	6.73	9.02	0.90	1.51	2.02	0.99	6.06	8.21	0.91	6.12	8.02	0.91	6.68	9.14	0.89	5.48	7.32	0.93	6.24	8.39	0.91	4.23	3.17	0.98	4.89	3.50	0.97
EDARADD+CCDC102B+FHL2	3	6.87	8.66	0.90	6.96	8.84	0.90	1.16	1.53	1.00	4.63	6.66	0.94	6.72	8.74	0.89	6.63	8.70	0.90	4.74	6.70	0.94	4.58	6.65	0.94	5.16	3.84	0.96	5.59	4.05	0.96
EDARADD+CCDC102B+C1orf132	3	6.56	8.11	0.91	6.80	9.30	0.89	1.28	1.61	1.00	5.54	8.14	0.92	6.48	8.20	0.91	6.74	9.49	0.89	5.18	7.04	0.93	5.71	8.52	0.91	4.62	3.67	0.97	5.38	3.92	0.97
EDARADD+CCDC102B+ASPA	3	8.32	10.45	0.85	8.80	10.99	0.85	1.37	1.79	1.00	5.80	8.49	0.91	8.20	10.63	0.85	8.83	11.35	0.84	5.70	7.85	0.92	6.30	8.83	0.90	6.35	4.71	0.95	7.15	5.20	0.94
EDARADD+FHL2+C1orf132	3	5.91	7.53	0.92	5.89	8.05	0.92	1.32	1.74	1.00	5.90	8.41	0.91	5.84	7.59	0.92	5.66	7.96	0.92	5.02	6.85	0.94	5.31	7.76	0.92	4.34	3.38	0.97	4.57	3.34	0.97
EDARADD+FHL2+ASPA	3	6.28	8.19	0.91	6.19	8.09	0.92	1.38	1.76	1.00	5.88	8.02	0.92	6.18	8.27	0.91	5.94	7.93	0.92	5.17	7.16	0.93	5.42	7.70	0.93	4.50	3.42	0.97	4.31	3.34	0.98
EDARADD+C1orf132+ASPA	3	6.54	8.35	0.90	7.04	9.68	0.88	1.52	1.96	0.99	6.76	9.21	0.89	6.47	8.43	0.90	7.01	9.83	0.88	5.66	7.65	0.92	6.52	9.35	0.89	4.79	3.70	0.97	5.56	4.01	0.96
CCDC102B+FHL2+C1orf132	3	5.77	7.39	0.92	5.66	7.78	0.92	1.24	1.61	1.00	5.15	7.56	0.93	5.69	7.44	0.92	5.46	7.73	0.93	4.61	6.45	0.94	4.58	6.75	0.94	4.05	3.11	0.98	4.14	2.95	0.98
CCDC102B+FHL2+ASPA	3	6.72	8.66	0.90	6.33	8.15	0.92	1.25	1.71	1.00	4.63	6.40	0.95	6.65	8.74	0.90	6.11	8.09	0.92	4.81	6.82	0.94	4.53	6.71	0.94	4.74	3.57	0.97	4.46	3.40	0.98
CCDC102B+C1orf132+ASPA	3	6.29	8.01	0.91	6.79	9.26	0.89	1.31	1.74	1.00	6.00	8.39	0.91	6.15	8.14	0.91	6.70	9.47	0.89	5.18	7.10	0.93	5.55	8.25	0.91	4.25	3.21	0.98	5.05	3.61	0.97
FHL2+C1orf132+ASPA	3	6.11	8.00	0.91	6.34	8.41	0.91	1.38	1.86	1.00	6.29	8.87	0.90	6.00	8.08	0.91	6.23	8.33	0.91	5.30	7.15	0.93	5.73	7.95	0.92	4.77	3.53	0.97	4.85	3.39	0.97
ELOVL2+EDARADD+CCDC102B+FHL2	4	6.46	8.30	0.90	6.88	8.67	0.90	0.78	1.07	1.00	4.83	6.79	0.94	6.37	8.36	0.90	6.72	8.63	0.91	4.37	6.19	0.95	4.76	6.91	0.94	5.27	3.83	0.96	5.75	4.43	0.96
ELOVL2+EDARADD+CCDC102B+C1orf132	4	6.03	7.60	0.92	6.18	8.47	0.91	0.85	1.12	1.00	5.11	7.38	0.93	6.03	7.63	0.92	6.15	8.58	0.91	4.52	6.33	0.94	5.54	7.68	0.93	4.48	3.42	0.97	5.09	3.83	0.97
ELOVL2+EDARADD+CCDC102B+ASPA	4	7.38	9.43	0.88	7.75	9.95	0.87	0.91	1.21	1.00	5.61	7.70	0.93	7.23	9.49	0.88	7.64	10.14	0.87	4.92	6.75	0.94	5.90	8.10	0.92	6.01	4.34	0.95	6.69	4.92	0.95
ELOVL2+EDARADD+FHL2+C1orf132	4	5.49	7.08	0.93	5.63	7.62	0.93	0.89	1.18	1.00	5.75	7.93	0.92	5.46	7.13	0.93	5.46	7.60	0.93	4.53	6.18	0.95	5.41	7.58	0.93	4.11	3.21	0.98	4.35	3.28	0.98
ELOVL2+EDARADD+FHL2+ASPA	4	6.00	7.84	0.91	6.21	7.97	0.92	0.92	1.19	1.00	5.76	7.67	0.93	5.98	7.91	0.91	6.03	7.91	0.92	4.63	6.45	0.94	5.41	7.62	0.93	4.49	3.41	0.97	4.52	3.55	0.97
ELOVL2+EDARADD+C1orf132+ASPA	4	5.82	7.50	0.92	6.19	8.49	0.91	0.97	1.32	1.00	5.72	7.71	0.92	5.79	7.58	0.92	6.14	8.61	0.91	4.92	6.75	0.94	5.66	8.00	0.92	4.23	3.23	0.98	4.85	3.54	0.97
ELOVL2+CCDC102B+FHL2+C1orf132	4	5.51	7.16	0.93	5.29	7.52	0.93	0.88	1.19	1.00	5.01	7.27	0.93	5.46	7.21	0.93	5.23	7.48	0.93	4.27	6.10	0.95	4.56	6.91	0.94	4.09	3.17	0.98	4.18	3.05	0.98
ELOVL2+CCDC102B+FHL2+ASPA	4	6.47	8.40	0.90	6.36	8.06	0.92	0.96	1.30	1.00	4.70	6.61	0.95	6.42	8.41	0.90	6.30	8.06	0.92	4.56	6.45	0.94	4.99	7.13	0.94	4.98	3.75	0.97	4.90	3.87	0.97
ELOVL2+CCDC102B+C1orf132+ASPA	4	5.86	7.59	0.92	6.13	8.51	0.91	0.94	1.26	1.00	5.09	7.42	0.93	5.81	7.71	0.92	6.12	8.76	0.91	4.62	6.51	0.94	5.36	7.53	0.93	4.15	3.14	0.98	4.72	3.51	0.97
ELOVL2+FHL2+C1orf132+ASPA	4	5.55	7.33	0.93	5.65	7.74	0.93	1.04	1.38	1.00	5.87	8.12	0.92	5.47	7.37	0.93	5.55	7.77	0.92	4.76	6.50	0.94	5.33	7.41	0.93	4.31	3.22	0.97	4.38	3.07	0.98
EDARADD+CCDC102B+FHL2+C1orf132	4	5.79	7.31	0.93	5.66	7.77	0.92	0.89	1.16	1.00	4.76	7.23	0.93	5.68	7.37	0.93	5.43	7.75	0.92	4.41	6.18	0.95	4.54	6.74	0.94	4.39	3.43	0.97	4.72	3.48	0.97
EDARADD+CCDC102B+FHL2+ASPA	4	6.30	8.17	0.91	6.22	8.04	0.92	0.89	1.21	1.00	4.54	6.63	0.94	6.16	8.28	0.91	5.97	7.94	0.92	4.55	6.41	0.94	4.41	6.71	0.94	4.94	3.68	0.97	5.08	3.74	0.97
EDARADD+CCDC102B+C1orf132+ASPA	4	6.27	7.93	0.91	6.67	9.14	0.90	0.97	1.26	1.00	5.49	7.91	0.92	6.13	8.05	0.91	6.65	9.38	0.89	4.99	6.87	0.93	5.57	8.21	0.92	4.74	3.72	0.97	5.50	4.09	0.96
EDARADD+FHL2+C1orf132+ASPA	4	5.67	7.40	0.92	5.74	7.85	0.92	1.03	1.36	1.00	5.79	8.28	0.91	5.58	7.48	0.92	5.66	7.79	0.92	4.74	6.59	0.94	5.23	7.55	0.93	4.48	3.35	0.97	4.68	3.34	0.97
CCDC102B+FHL2+C1orf132+ASPA	4	5.60	7.30	0.93	5.55	7.65	0.93	0.92	1.24	1.00	5.02	7.36	0.93	5.49	7.39	0.93	5.37	7.59	0.93	4.45	6.24	0.95	4.44	6.67	0.94	4.27	3.14	0.98	4.35	3.01	0.98
ELOVL2+EDARADD+CCDC102B+FHL2+C1orf132	5	5.47	7.05	0.93	5.47	7.53	0.93	0.63	0.86	1.00	4.71	7.05	0.94	5.43	7.10	0.93	5.32	7.49	0.93	4.09	5.80	0.95	4.62	6.99	0.94	4.36	3.36	0.97	4.70	3.58	0.97
ELOVL2+EDARADD+CCDC102B+FHL2+ASPA	5	6.01	7.85	0.91	6.21	7.97	0.92	0.67	0.90	1.00	4.88	6.82	0.94	5.96	7.88	0.91	5.99	7.93	0.92	4.29	6.05	0.95	4.81	7.03	0.94	4.99	3.65	0.97	5.26	4.01	0.97
ELOVL2+EDARADD+CCDC102B+C1orf132+ASPA	5	5.77	7.45	0.92	6.06	8.39	0.91	0.68	0.91	1.00	5.15	7.25	0.93	5.76	7.60	0.92	6.03	8.62	0.91	4.45	6.30	0.95	5.43	7.58	0.93	4.54	3.43	0.97	5.18	3.87	0.97
ELOVL2+EDARADD+FHL2+C1orf132+ASPA	5	5.32	6.98	0.93	5.43	7.50	0.93	0.75	0.98	1.00	5.49	7.77	0.92	5.25	7.05	0.93	5.30	7.48	0.93	4.35	6.04	0.95	5.11	7.30	0.93	4.23	3.21	0.98	4.45	3.24	0.98
ELOVL2+CCDC102B+FHL2+C1orf132+ASPA	5	5.37	7.08	0.93	5.27	7.43	0.93	0.74	1.00	1.00	4.90	7.15	0.94	5.31	7.14	0.93	5.20	7.48	0.93	4.21	5.92	0.95	4.60	6.80	0.94	4.24	3.18	0.98	4.34	3.11	0.98
EDARADD+CCDC102B+FHL2+C1orf132+ASPA	5	5.59	7.22	0.93	5.57	7.62	0.93	0.73	0.94	1.00	4.62	7.03	0.94	5.43	7.31	0.93	5.34	7.58	0.93	4.29	6.01	0.95	4.39	6.68	0.94	4.50	3.40	0.97	4.77	3.49	0.97
ELOVL2+EDARADD+CCDC102B+FHL2+C1orf132+ASPA	6	5.30	6.96	0.93	5.33	7.43	0.93	0.54	0.72	1.00	4.47	6.85	0.94	5.23	7.04	0.93	5.21	7.46	0.93	4.02	5.70	0.96	4.54	6.86	0.94	4.43	3.32	0.97	4.72	3.51	0.97

Table S7: Age prediction performances of the different epigenetic clocks based on different CpG combinations using transformed and standardized DNA methylation data.

CpG combination	CpG nb.	ENR						GBR						SVM_l						SVM_r						KDR					
		Training set			Testing set			Training set			Testing set			Training set			Testing set			Training set			Testing set			Training set			Testing set		
		MAE	RMSE	R^2	MAE	RMSE	R^2	MAE	RMSE	R^2	MAE	RMSE	R^2	MAE	RMSE	R^2	MAE	RMSE	R^2	MAE	RMSE	R^2	MAE	RMSE	R^2	MAE	RMSE	R^2	MAE	RMSE	R^2
ELOVL2+EDARADD	2	7.16	9.14	0.88	7.67	10.52	0.86	1.99	2.60	0.99	6.00	8.72	0.90	7.15	9.15	0.88	7.79	10.66	0.86	5.90	8.14	0.91	6.74	9.50	0.89	3.25	4.23	0.98	3.86	5.07	0.97
ELOVL2+CCDC102B	2	7.50	9.61	0.87	7.41	10.04	0.88	1.88	2.57	0.99	5.82	7.83	0.92	7.40	9.67	0.87	7.60	10.41	0.88	5.63	7.69	0.92	6.73	8.98	0.90	3.76	5.04	0.97	4.36	5.85	0.97
ELOVL2+FHL2	2	6.83	9.14	0.88	7.03	9.08	0.90	2.12	2.80	0.99	7.20	9.38	0.89	6.80	9.14	0.88	6.94	9.04	0.90	5.93	8.38	0.90	6.53	8.91	0.90	3.68	4.89	0.97	3.65	4.71	0.97
ELOVL2+C1orf132	2	6.55	8.19	0.91	6.73	9.07	0.90	2.06	2.70	0.99	6.69	8.87	0.90	6.50	8.21	0.91	6.60	9.11	0.90	6.09	7.88	0.91	6.34	8.68	0.91	3.10	3.98	0.98	3.14	4.42	0.98
ELOVL2+ASPA	2	8.32	10.95	0.83	8.74	11.43	0.83	2.51	3.28	0.99	7.52	10.24	0.87	8.24	11.02	0.83	8.68	11.68	0.83	6.92	9.20	0.88	7.47	9.79	0.88	4.20	5.58	0.96	4.21	5.79	0.96
EDARADD+CCDC102B	2	7.91	10.12	0.86	7.74	10.09	0.88	1.83	2.42	0.99	5.79	8.49	0.91	7.71	10.27	0.86	7.76	10.54	0.88	6.09	8.36	0.90	6.13	8.76	0.90	4.34	5.96	0.95	4.63	6.61	0.96
EDARADD+FHL2	2	6.53	8.43	0.90	6.62	8.90	0.90	1.94	2.50	0.99	5.96	8.29	0.91	6.47	8.46	0.90	6.39	8.78	0.90	5.60	7.58	0.92	5.78	8.41	0.91	3.32	4.33	0.97	3.24	4.52	0.97
EDARADD+C1orf132	2	7.24	8.90	0.89	7.89	10.37	0.86	2.11	2.64	0.99	6.99	9.72	0.88	7.15	9.02	0.89	7.78	10.46	0.87	6.34	8.14	0.91	6.83	9.85	0.88	3.86	5.00	0.97	4.26	5.83	0.96
EDARADD+ASPA	2	8.27	10.76	0.84	8.60	11.12	0.84	2.48	3.10	0.99	8.09	10.56	0.86	8.20	10.90	0.84	8.50	11.22	0.84	7.07	9.35	0.88	7.77	10.62	0.86	4.32	5.86	0.95	4.49	6.05	0.96
CCDC102B+FHL2	2	6.17	8.38	0.90	5.78	7.93	0.92	1.80	2.45	0.99	4.66	6.55	0.95	6.13	8.44	0.90	5.80	8.14	0.92	4.97	7.06	0.93	4.32	6.18	0.95	3.19	4.46	0.97	3.08	4.43	0.98
CCDC102B+C1orf132	2	6.14	8.00	0.91	6.21	8.97	0.90	1.79	2.31	0.99	5.95	8.45	0.91	6.01	8.14	0.91	6.17	9.12	0.90	5.33	7.30	0.93	5.68	8.32	0.91	3.09	4.15	0.98	3.32	4.90	0.97
CCDC102B+ASPA	2	7.82	10.53	0.85	7.33	10.52	0.86	1.97	2.70	0.99	6.08	8.87	0.90	7.79	10.58	0.85	7.45	10.83	0.86	6.18	8.43	0.90	6.33	9.20	0.89	4.27	5.96	0.95	4.30	6.58	0.95
FHL2+C1orf132	2	6.39	8.19	0.91	6.49	8.65	0.91	1.83	2.48	0.99	6.83	9.49	0.89	6.31	8.22	0.91	6.32	8.52	0.91	5.67	7.54	0.92	6.04	8.35	0.91	3.55	4.58	0.97	3.37	4.64	0.97
FHL2+ASPA	2	7.18	9.33	0.88	6.74	8.67	0.91	2.19	2.78	0.99	6.71	8.67	0.91	7.10	9.37	0.88	6.48	8.48	0.91	6.13	8.33	0.90	6.09	8.12	0.92	3.64	4.97	0.97	3.16	4.26	0.98
C1orf132+ASPA	2	7.39	9.41	0.88	8.59	11.45	0.83	2.33	3.02	0.99	7.45	10.20	0.87	7.30	9.50	0.88	8.47	11.51	0.83	6.35	8.48	0.90	7.37	10.29	0.87	4.02	5.26	0.96	4.67	6.33	0.95
ELOVL2+EDARADD+CCDC102B	3	6.71	8.48	0.90	7.16	9.54	0.89	1.17	1.53	1.00	5.27	7.40	0.93	6.67	8.55	0.90	7.34	9.79	0.89	4.98	6.85	0.94	6.17	8.45	0.91	3.65	4.81	0.97	4.22	5.78	0.96
ELOVL2+EDARADD+FHL2	3	5.82	7.53	0.92	6.44	8.59	0.91	1.14	1.52	1.00	5.81	7.90	0.92	5.81	7.55	0.92	6.41	8.52	0.91	4.79	6.71	0.94	5.86	8.14	0.92	3.00	3.91	0.98	3.29	4.39	0.98
ELOVL2+EDARADD+C1orf132	3	5.78	7.42	0.92	6.48	8.57	0.91	1.19	1.60	1.00	5.86	8.01	0.92	5.77	7.44	0.92	6.43	8.61	0.91	5.21	6.95	0.93	5.94	8.13	0.92	2.99	3.91	0.98	3.40	4.64	0.97
ELOVL2+EDARADD+ASPA	3	6.65	8.60	0.90	6.98	9.62	0.88	1.34	1.76	1.00	6.54	8.69	0.90	6.55	8.70	0.90	6.99	9.66	0.88	5.31	7.28	0.93	6.09	8.56	0.91	3.30	4.42	0.97	3.61	4.98	0.97
ELOVL2+CCDC102B+FHL2	3	5.84	7.87	0.91	6.07	8.01	0.92	1.21	1.67	1.00	4.79	6.75	0.94	5.80	7.91	0.91	5.93	8.10	0.92	4.60	6.57	0.94	4.97	6.98	0.94	3.21	4.40	0.97	3.44	4.71	0.97
ELOVL2+CCDC102B+C1orf132	3	5.38	7.20	0.93	5.56	8.11	0.92	1.16	1.53	1.00	5.31	7.56	0.93	5.36	7.22	0.93	5.59	8.17	0.92	4.65	6.56	0.94	5.66	7.99	0.92	2.79	3.74	0.98	3.04	4.42	0.98
ELOVL2+CCDC102B+ASPA	3	6.93	9.13	0.88	6.95	9.69	0.89	1.38	1.91	0.99	5.82	7.96	0.92	6.85	9.20	0.88	7.01	9.99	0.88	5.23	7.23	0.93	6.36	8.70	0.91	3.84	5.19	0.96	4.15	5.96	0.96
ELOVL2+FHL2+C1orf132	3	5.71	7.42	0.92	5.69	7.82	0.92	1.27	1.71	1.00	6.47	8.65	0.91	5.67	7.48	0.92	5.55	7.73	0.93	5.07	6.86	0.93	5.68	7.77	0.92	3.17	4.16	0.98	3.01	4.21	0.98
ELOVL2+FHL2+ASPA	3	6.39	8.50	0.90	6.41	8.33	0.91	1.45	1.92	1.00	6.12	8.28	0.91	6.37	8.50	0.90	6.23	8.26	0.91	5.42	7.37	0.93	5.68	7.92	0.92	3.54	4.71	0.97	3.28	4.44	0.98
ELOVL2+C1orf132+ASPA	3	6.24	7.98	0.91	6.67	9.04	0.90	1.53	2.05	0.99	6.07	8.18	0.92	6.12	8.05	0.91	6.66	9.18	0.89	5.45	7.30	0.93	6.15	8.29	0.91	3.19	4.32	0.97	3.55	4.97	0.97
EDARADD+CCDC102B+FHL2	3	6.01	7.88	0.91	5.85	7.92	0.92	1.16	1.54	1.00	4.53	6.57	0.95	5.84	7.97	0.91	5.60	7.84	0.92	4.64	6.58	0.94	4.33	6.46	0.95	3.35	4.59	0.97	3.42	4.88	0.97
EDARADD+CCDC102B+C1orf132	3	6.10	7.89	0.91	6.13	8.80	0.90	1.27	1.61	1.00	5.57	8.09	0.92	5.98	8.00	0.91	6.07	8.96	0.91	5.07	6.95	0.93	5.59	8.48	0.91	3.58	4.75	0.97	3.90	5.46	0.97
EDARADD+CCDC102B+ASPA	3	7.25	9.64	0.87	7.14	9.64	0.88	1.38	1.81	1.00	5.84	8.53	0.91	7.11	9.77	0.87	7.34	9.98	0.88	5.57	7.68	0.92	6.42	9.18	0.90	4.28	6.02	0.95	4.67	6.46	0.96
EDARADD+FHL2+C1orf132	3	5.98	7.59	0.92	6.12	8.18	0.92	1.30	1.73	1.00	5.99	8.45	0.91	5.89	7.64	0.92	5.85	8.08	0.92	5.02	6.84	0.94	5.48	7.95	0.92	3.41	4.46	0.97	3.50	4.78	0.97
EDARADD+FHL2+ASPA	3	6.19	8.13	0.91	6.19	8.14	0.92	1.36	1.74	1.00	5.94	8.06	0.92	6.06	8.20	0.91	5.92	7.93	0.92	5.14	7.08	0.93	5.37	7.78	0.92	3.29	4.51	0.97	3.25	4.33	0.98
EDARADD+C1orf132+ASPA	3	6.88	8.67	0.90	7.59	10.05	0.87	1.49	1.93	0.99	6.81	9.28	0.89	6.77	8.82	0.90	7.47	10.13	0.87	5.63	7.59	0.92	6.43	9.24	0.89	3.96	5.23	0.96	4.40	5.99	0.96
CCDC102B+FHL2+C1orf132	3	5.34	7.22	0.93	5.20	7.45	0.93	1.24	1.60	1.00	5.16	7.62	0.93	5.23	7.27	0.93	5.00	7.50	0.93	4.55	6.36	0.94	4.32	6.52	0.95	3.00	4.13	0.98	2.88	4.31	0.98
CCDC102B+FHL2+ASPA	3	6.05	8.20	0.91	5.39	7.66	0.93	1.24	1.70	1.00	4.59	6.32	0.95	6.02	8.25	0.91	5.41	7.79	0.93	4.74	6.70	0.94	4.47	6.81	0.94	3.41	4.74	0.97	3.12	4.59	0.98
CCDC102B+C1orf132+ASPA	3	6.10	7.97	0.91	6.23	8.99	0.90	1.31	1.73	1.00	6.00	8.37	0.91	5.93	8.10	0.91	6.16	9.17	0.90	5.15	7.04	0.93	5.62	8.26	0.91	3.33	4.66	0.97	3.62	5.47	0.97
FHL2+C1orf132+ASPA	3	6.11	8.00	0.91	6.34	8.41	0.91	1.38	1.87	1.00	6.24	8.79	0.90	6.00	8.08	0.91	6.23	8.33	0.91	5.30	7.15	0.93	5.73	7.95	0.92	3.53	4.77	0.97	3.39	4.85	0.97
ELOVL2+EDARADD+CCDC102B+FHL2	4	5.55	7.26	0.93	6.11	8.05	0.92	0.80	1.09	1.00	4.74	6.74	0.94	5.50	7.34	0.93	5.89	7.92	0.92	4.29	6.16	0.95	4.92	7.04	0.94	3.13	4.24	0.98	3.51	4.85	0.97
ELOVL2+EDARADD+CCDC102B+C1orf132	4	5.34	7.01	0.93	5.78	8.03	0.92	0.85	1.12	1.00	5.00	7.23	0.93	5.32	7.03	0.93	5.75	8.07	0.92	4.42	6.20	0.95	5.88	7.95	0.92	3.04	4.08	0.98	3.49	4.87	0.97
ELOVL2+EDARADD+CCDC102B+ASPA	4	6.38	8.23	0.91	6.78	9.14	0.90	0.90	1.20	1.00	5.65	7.66	0.93	6.30	8.34	0.90	6.69	9.19	0.90	4.77	6.63	0.94	6.09	8.39	0.91	3.66	4.98	0.97	4.13	5.76	0.96
ELOVL2+EDARADD+FHL2+C1orf132	4	5.24	6.84	0.94	5.70	7.69	0.93	0.89	1.18	1.00	5.65	7.83	0.92	5.22	6.85	0.94	5.69	7.68	0.93	4.53	6.17	0.95	5.52	7.60	0.93	2.98	3.94	0.98	3.15	4.35	0.98
ELOVL2+EDARADD+FHL2+ASPA	4	5.58	7.35	0.93	6.05	8.07	0.92	0.91	1.19	1.00	5.71	7.62	0.93	5.57	7.38	0.92	5.93	7.94	0.92	4.60	6.42	0.94	5.32	7.49	0.93	3.08	4.08	0.98	3.14	4.28	0.98
ELOVL2+EDARADD+C1orf132+ASPA	4	5.65	7.34	0.93	6.25	8.46	0.91	0.97	1.31	1.00	5.77	7.82	0.92	5.57	7.42	0.92	6.09	8.57	0.91	4.81	6.64	0.94	5.64	7.95	0.92	3.10	4.21	0.98	3.49	4.92	0.97
ELOVL2+CCDC102B+FHL2+C1orf132	4	5.03	6.77	0.94	4.92	7.26	0.93	0.86	1.17	1.00	5.03	7.27	0.93	4.99	6.83	0.94	4.80	7.23	0.93	4.19	6.00	0.95	4.69	6.98	0.94	2.91	3.91	0.98	2.81	4.21	0.98
ELOVL2+CCDC102B+FHL2+ASPA	4	5.72	7.71	0.92	5.81	7.77	0.92	0.96	1.31	1.00	4.71	6.64	0.94	5.69	7.74	0.92	5.70	7.80	0.92	4.52	6.43	0.94	5.01	7.26	0.93	3.34	4.57	0.97	3.40	4.77	0.97
ELOVL2+CCDC102B+C1orf132+ASPA	4	5.36	7.19	0.93	5.58	8.13	0.92	0.94	1.27	1.00	5.08	7.38	0.93	5.32	7.27	0.93	5.60	8.27	0.92	4.49	6.45	0.94	5.55	7.79	0.92	3.00	4.15	0.98	3.30	4.90	0.97
ELOVL2+FHL2+C1orf132+ASPA	4	5.54	7.31	0.93	5.55	7.74	0.92	1.05	1.39	1.00	5.88	8.13	0.92	5.48	7.36	0.93	5.47	7.76	0.92	4.74	6.46	0.94	5.24	7.34	0.93	3.23	4.33	0.97	3.07	4.44	0.98
EDARADD+CCDC102B+FHL2+C1orf132	4	5.36	7.11	0.93	5.24	7.41	0.93	0.88	1.15	1.00	4.77	7.30	0.93	5.20	7.18	0.93	4.92	7.39	0.93	4.37	6.10	0.95	4.32	6.64	0.94	3.25	4.40	0.97	3.23	4.76	0.97
EDARADD+CCDC102B+FHL2+ASPA	4	5.81	7.75	0.92	5.59	7.61	0.93	0.89	1.20	1.00	4.54	6.63	0.94	5.72	7.85	0.92	5.39	7.57	0.93	4.45	6.31	0.94	4.58	6.93	0.94	3.44	4.80	0.97	3.46	4.88	0.97
EDARADD+CCDC102B+C1orf132+ASPA	4	6.07	7.87	0.91	6.16	8.76	0.90	0.99	1.27	1.00	5.56	8.05	0.92	5.89	8.03	0.91	6.10	9.05	0.90	4.91	6.77	0.94	5.70	8.46	0.91	3.71	5.05	0.97	4.05	5.73	0.96
EDARADD+FHL2+C1orf132+ASPA	4	5.81	7.50	0.92	6.00	7.95	0.92	1.04	1.36	1.00	5.87	8.24	0.91	5.68	7.57	0.92	5.80	7.79	0.92	4.69	6.54	0.94	5.26	7.64	0.93	3.46	4.66	0.97	3.56	4.88	0.97
CCDC102B+FHL2+C1orf132+ASPA	4	5.33	7.21	0.93	5.17	7.43	0.93	0.93	1.24	1.00	5.05	7.41	0.93	5.19	7.25	0.93	4.95	7.46	0.93	4.40	6.18	0.95	4.42	6.73	0.94	3.14	4.44	0.97	2.96	4.63	0.97
ELOVL2+EDARADD+CCDC102B+FHL2+C1orf132	5	4.95	6.59	0.94	5.22	7.30	0.93	0.62	0.85	1.00	4.70	7.04	0.94	4.93	6.66	0.94	5.00	7.27	0.93	4.01	5.76	0.95	4.80	7.04	0.94	2.99	4.03	0.98	3.15	4.55	0.98
ELOVL2+EDARADD+CCDC102B+FHL2+ASPA	5	5.43	7.17	0.93	5.85	7.78	0.92	0.66	0.90	1.00	4.83	6.75	0.94	5.39	7.22	0.93	5.64	7.65	0.93	4.20	5.97	0.95	4.94	7.17	0.94	3.23	4.42	0.97	3.40	4.84	0.97
ELOVL2+EDARADD+CCDC102B+C1orf132+ASPA	5	5.31	7.00	0.93	5.75	8.02	0.92	0.69	0.92	1.00	5.05	7.21	0.93	5.26	7.03	0.93	5.68	8.11	0.92	4.35	6.15	0.95	5.71	7.86	0.92	3.17	4.37	0.97	3.63	5.14	0.97
ELOVL2+EDARADD+FHL2+C1orf132+ASPA	5	5.15	6.80	0.94	5.55	7.57	0.93	0.75	0.99	1.00	5.45	7.73	0.92	5.10	6.85	0.94	5.31	7.49	0.93	4.33	6.00	0.95	5.06	7.23	0.93	3.11	4.14	0.98	3.16	4.48	0.97
ELOVL2+CCDC102B+FHL2+C1orf132+ASPA	5	5.00	6.76	0.94	4.90	7.26	0.93	0.73	0.99	1.00	4.94	7.23	0.93	4.94	6.81	0.94	4.79	7.27	0.93	4.16	5.89	0.95	4.65	6.97	0.94	3.00	4.17	0.98	2.89	4.48	0.98
EDARADD+CCDC102B+FHL2+C1orf132+ASPA	5	5.32	7.10	0.93	5.21	7.38	0.93	0.75	0.96	1.00	4.60	6.99	0.94	5.13	7.19	0.93	4.85	7.35	0.93	4.18	5.90	0.95	4.54	6.92	0.94	3.34	4.63	0.97	3.34	4.92	0.97
ELOVL2+EDARADD+CCDC102B+FHL2+C1orf132+ASPA	6	4.93	6.59	0.94	5.18	7.27	0.93	0.53	0.73	1.00	4.46	6.87	0.94	4.88	6.65	0.94	4.90	7.22	0.93	3.95	5.64	0.96	4.73	7.01	0.94	3.08	4.25	0.98	3.18	4.71	0.97

References

1. Ferreira JP, Girerd N, Bozec E, Merckle L, Pizard A, Bouali S, et al. Cohort Profile: Rationale and design of the fourth visit of the STANISLAS cohort: a familial longitudinal population-based cohort from the Nancy region of France. Int J Epidemiol. 2018;47(2):395-j.
2. How-Kit A, Sahbatou M, Hardy LM, Tessier NP, Schiavon V, Le Buanec H, et al. The CEPH aging cohort and biobank: a valuable collection of biological samples from exceptionally long-lived French individuals and their offspring for longevity studies. Geroscience. 2024;46(2):2681-95.
3. Daunay A, Hardy L, Bouyacoub Y, Sahbatou M, Touvier M., Blanché H, Deleuze JF, How-Kit A. Centenarians consistently present a younger epigenetic age than their chronological age with four epigenetic clocks based on a small number of CpG sites Aging (Milano). 2022.
4. Campan M, Weisenberger DJ, Trinh B, Laird PW. MethyLight. Methods Mol Biol. 2009;507:325-37.
5. How-Kit A, Daunay A, Mazaleyrat N, Busato F, Daviaud C, Teyssier E, et al. Accurate CpG and non-CpG cytosine methylation analysis by high-throughput locus-specific pyrosequencing in plants. Plant Mol Biol. 2015;88(4-5):471-85.
6. How-Kit A, Tost J. Pyrosequencing(R)-Based Identification of Low-Frequency Mutations Enriched Through Enhanced-ice-COLD-PCR. Methods Mol Biol. 2015;1315:83-101.
7. Zhou H, Hastie T. Regularization and variable selection via the elastic net. J R Stat Soc Ser B (Stat Methodol). 2005;67(2):301–20.
8. Friedman JH. Greedy function approximation: A gradient boosting machine. The Annals of Statistics. 2001;29(5):1189-232.
9. Du K-L, Jiang B, Lu D, Hua J, Swamy MNS. Exploring kernel machines and support vector machines: principles, techniques, and future directions. Mathematics. 2024;12(24):3935.
10. Klemera P, Doubal S. A new approach to the concept and computation of biological age. Mech Ageing Dev. 2006;127(3):240-8.
11. Horvath S, Raj K. DNA methylation-based biomarkers and the epigenetic clock theory of ageing. Nature reviews Genetics. 2018;19(6):371-84.
12. Teschendorff AE, Horvath S. Epigenetic ageing clocks: statistical methods and emerging computational challenges. Nat Rev Genet. 2025;26(5):350-68.
13. Garali I, Sahbatou M, Daunay A, Baudrin LG, Renault V, Bouyacoub Y, et al. Improvements and inter-laboratory implementation and optimization of blood-based single-locus age prediction models using DNA methylation of the ELOVL2 promoter. Sci Rep. 2020;10(1):15652.
14. Levine ME. Modeling the rate of senescence: can estimated biological age predict mortality more accurately than chronological age? J Gerontol A Biol Sci Med Sci. 2013;68(6):667-74.
15. Horvath S. DNA methylation age of human tissues and cell types. Genome Biol. 2013;14(10):R115.
16. Alisch RS, Barwick BG, Chopra P, Myrick LK, Satten GA, Conneely KN, et al. Age-associated DNA methylation in pediatric populations. Genome Res. 2012;22(4):623-32.
17. Carlsen L, Hollander O, Danzer MF, Vennemann M, Augustin C. DNA methylation-based age estimation for adults and minors: considering sex-specific differences and non-linear correlations. Int J Legal Med. 2023;137(3):635-43.
18. Jones MJ, Konwar C, Asiimwe R, Dinh L, Razzaghian HR, de Goede O, et al. DNA methylation differences between cord and adult white blood cells reflect postnatal immune cell maturation. Commun Biol. 2025;8(1):237.
19. Zhang Z, Reynolds SR, Stolrow HG, Chen JQ, Christensen BC, Salas LA. Deciphering the role of immune cell composition in epigenetic age acceleration: Insights from cell-type deconvolution applied to human blood epigenetic clocks. Aging Cell. 2024;23(3):e14071.
20. Daunay A, Baudrin LG, Deleuze JF, How-Kit A. Evaluation of six blood-based age prediction models using DNA methylation analysis by pyrosequencing. Sci Rep. 2019;9(1):8862.
21. Quantitative comparison of DNA methylation assays for biomarker development and clinical applications. Nat Biotechnol. 2016;34(7):726-37.
22. Freire-Aradas A, Pospiech E, Aliferi A, Giron-Santamaria L, Mosquera-Miguel A, Pisarek A, et al. A Comparison of Forensic Age Prediction Models Using Data From Four DNA Methylation Technologies. Front Genet. 2020;11:932.
23. Hong SR, Shin KJ, Jung SE, Lee EH, Lee HY. Platform-independent models for age prediction using DNA methylation data. Forensic Sci Int Genet. 2019;38:39-47.

24. Dias HC, Cordeiro C, Pereira J, Pinto C, Real FC, Cunha E, et al. DNA methylation age estimation in blood samples of living and deceased individuals using a multiplex SNaPshot assay. Forensic Sci Int. 2020;311:110267.
25. Jung SE, Lim SM, Hong SR, Lee EH, Shin KJ, Lee HY. DNA methylation of the ELOVL2, FHL2, KLF14, C1orf132/MIR29B2C, and TRIM59 genes for age prediction from blood, saliva, and buccal swab samples. Forensic Sci Int Genet. 2019;38:1-8.
26. Han Y, Franzen J, Stiehl T, Gobs M, Kuo CC, Nikolic M, et al. New targeted approaches for epigenetic age predictions. BMC Biol. 2020;18(1):71.
27. Gensous N, Sala C, Pirazzini C, Ravaioli F, Milazzo M, Kwiatkowska KM, et al. A Targeted Epigenetic Clock for the Prediction of Biological Age. Cells. 2022;11(24).
28. Garagnani P, Bacalini MG, Pirazzini C, Gori D, Giuliani C, Mari D, et al. Methylation of ELOVL2 gene as a new epigenetic marker of age. Aging cell. 2012;11(6):1132-4.
29. Suchiman HE, Slieker RC, Kremer D, Slagboom PE, Heijmans BT, Tobi EW. Design, measurement and processing of region-specific DNA methylation assays: the mass spectrometry-based method EpiTYPER. Front Genet. 2015;6:287.
30. Ho Lee M, Hee Hwang J, Min Seong K, Jin Ahn J, Jun Kim S, Yong Hwang S, et al. Application of droplet digital PCR method for DNA methylation-based age prediction from saliva. Leg Med (Tokyo). 2022;54:101992.
31. Dias HC, Manco L. Predicting age from blood by droplet digital PCR using a set of three DNA methylation markers. Forensic Sci Int. 2024;356:111950.
32. Zhou Y, Wang Y, Song M, Jiang L, Sun C, Wang S, et al. A high-throughput droplet digital PCR system aiming eight DNA methylation targets for age prediction. J Pharm Biomed Anal. 2024;240:115943.
33. Yu M, Carter KT, Makar KW, Vickers K, Ulrich CM, Schoen RE, et al. MethyLight droplet digital PCR for detection and absolute quantification of infrequently methylated alleles. Epigenetics. 2015;10(9):803-9.
34. Wiencke JK, Bracci PM, Hsuang G, Zheng S, Hansen H, Wrensch MR, et al. A comparison of DNA methylation specific droplet digital PCR (ddPCR) and real time qPCR with flow cytometry in characterizing human T cells in peripheral blood. Epigenetics. 2014;9(10):1360-5.
35. Rutsaert S, Bosman K, Trypsteen W, Nijhuis M, Vandekerckhove L. Digital PCR as a tool to measure HIV persistence. Retrovirology. 2018;15(1):16.
36. Basu AS. Digital Assays Part I: Partitioning Statistics and Digital PCR. SLAS Technol. 2017;22(4):369-86.
37. Vidaki A, Ballard D, Aliferi A, Miller TH, Barron LP, Syndercombe Court D. DNA methylation-based forensic age prediction using artificial neural networks and next generation sequencing. Forensic Sci Int Genet. 2017;28:225-36.
38. Aliferi A, Ballard D, Gallidabino MD, Thurtle H, Barron L, Syndercombe Court D. DNA methylation-based age prediction using massively parallel sequencing data and multiple machine learning models. Forensic Sci Int Genet. 2018;37:215-26.
39. McEwen LM, Jones MJ, Lin DTS, Edgar RD, Husquin LT, MacIsaac JL, et al. Systematic evaluation of DNA methylation age estimation with common preprocessing methods and the Infinium MethylationEPIC BeadChip array. Clin Epigenetics. 2018;10(1):123.
40. Dhingra R, Kwee LC, Diaz-Sanchez D, Devlin RB, Cascio W, Hauser ER, et al. Evaluating DNA methylation age on the Illumina MethylationEPIC Bead Chip. PLoS One. 2019;14(4):e0207834.
41. Ori APS, Lu AT, Horvath S, Ophoff RA. Significant variation in the performance of DNA methylation predictors across data preprocessing and normalization strategies. Genome Biol. 2022;23(1):225.
42. Bibikova M, Barnes B, Tsan C, Ho V, Klotzle B, Le JM, et al. High density DNA methylation array with single CpG site resolution. Genomics. 2011;98(4):288-95.
43. Simpson DJ, Zhao Q, Olova NN, Dabrowski J, Xie X, Latorre-Crespo E, et al. Region-based epigenetic clock design improves RRBS-based age prediction. Aging Cell. 2023;22(8):e13866.
